# Intestinal microbiota imbalance resulted by anti-*Toxoplasma gondii* immune responses aggravate gut and brain injury

**DOI:** 10.1186/s13071-024-06349-8

**Published:** 2024-07-02

**Authors:** Jiating Chen, Chi Zhang, Zihan Yang, Weiling Wu, Weihao Zou, Zixuan Xin, Shuyu Zheng, Runchun Liu, Lili Yang, Hongjuan Peng

**Affiliations:** Department of Pathogen Biology, Guangdong Provincial Key Laboratory of Tropical Diseases Research, School of Public Health, Key Laboratory of Infectious Diseases Research in South China (Southern Medical University), Ministry of Education, Southern Medical University, 1023-1063 South Shatai Rd, Guangzhou, 510515 Guangdong People’s Republic of China

**Keywords:** *Toxoplasma gondii*, Neurodegenerative diseases, Intestinal dysbiosis, *Lactobacillus murinus*, *Lactobacillus gasseri*, Indole-3-lactic acid

## Abstract

**Background:**

*Toxoplasma gondii* infection affects a significant portion of the global population, leading to severe toxoplasmosis and, in immunocompromised patients, even death. During *T. gondii* infection, disruption of gut microbiota further exacerbates the damage to intestinal and brain barriers. Therefore, identifying imbalanced probiotics during infection and restoring their equilibrium can regulate the balance of gut microbiota metabolites, thereby alleviating tissue damage.

**Methods:**

Vimentin gene knockout (*vim−/−*) mice were employed as an immunocompromised model to evaluate the influence of host immune responses on gut microbiota balance during *T. gondii* infection. Behavioral experiments were performed to assess changes in cognitive levels and depressive tendencies between chronically infected *vim−/−* and wild-type (WT) mice. Fecal samples were subjected to 16S ribosomal RNA (rRNA) sequencing, and serum metabolites were analyzed to identify potential gut probiotics and their metabolites for the treatment of *T. gondii* infection.

**Results:**

Compared to the immunocompetent WT sv129 mice, the immunocompromised mice exhibited lower levels of neuronal apoptosis and fewer neurobehavioral abnormalities during chronic infection. 16S rRNA sequencing revealed a significant decrease in the abundance of probiotics, including several species of *Lactobacillus*, in WT mice. Restoring this balance through the administration of *Lactobacillus murinus* and *Lactobacillus gasseri* significantly suppressed the *T. gondii* burden in the intestine, liver, and brain. Moreover, transplantation of these two *Lactobacillus *spp. significantly improved intestinal barrier damage and alleviated inflammation and neuronal apoptosis in the central nervous system. Metabolite detection studies revealed that the levels of various *Lactobacillus*-related metabolites, including indole-3-lactic acid (ILA) in serum, decreased significantly after *T. gondii* infection. We confirmed that *L. gasseri* secreted much more ILA than *L. murinus*. Notably, ILA can activate the aromatic hydrocarbon receptor signaling pathway in intestinal epithelial cells, promoting the activation of CD8^+^ T cells and the secretion of interferon-gamma.

**Conclusion:**

Our study revealed that host immune responses against *T. gondii* infection severely disrupted the balance of gut microbiota, resulting in intestinal and brain damage. *Lactobacillus* spp. play a crucial role in immune regulation, and the metabolite ILA is a promising therapeutic compound for efficient and safe treatment of *T. gondii* infection.

**Graphical Abstract:**

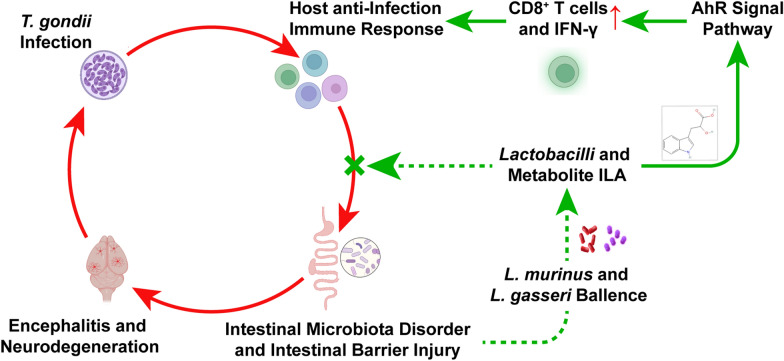

**Supplementary Information:**

The online version contains supplementary material available at 10.1186/s13071-024-06349-8.

## Background

*Toxoplasma gondii* is an opportunistic parasitic pathogen that causes toxoplasmosis in humans, with approximately one-third of the global population estimated to be infected [1, 2]. Although the infection is mostly asymptomatic in immunocompetent humans, it can lead to adverse pregnancy outcomes or mental retardation in newborns [3–5], while in severely immunocompromised patients, it may cause encephalitis, retinochoroiditis or even death [6]. High doses of oral *T. gondii* infection can result in fatal enteritis [7]. Once *T. gondii* damages intestinal epithelial cells (IECs), it directly impairs the integrity of the intestinal barrier, leading to intestinal leakage [8]. This, in turn, can cause intestinal dysbiosis and facilitate the systemic dissemination of *T. gondii* [9], with the potential consequence that *T. gondii* can establish persistent infection by forming cysts in tissues such as muscle, eyes and brain [10, 11]. Its persistent parasitism in the central nervous system (CNS) poses a significant risk for the development of anxiety and exacerbation of other neurological and psychiatric abnormalities [6, 12].

Similar to *T. gondii*, Zika virus (ZIKV) and human immunodeficiency virus (HIV) can invade the CNS and induce neuronal apoptosis, contributing to neurodegeneration [13, 14]. Specifically, ZIKV exhibits a tropism for neural progenitor cells, leading to neuronal apoptosis and disruption of embryonic brain growth, ultimately resulting in the development of microcephaly [15, 16]. HIV and *T. gondii* exploit macrophages or dendritic cells (DCs) to breach the blood–brain barrier (BBB) [17], inducing the release of cytokines (including tumor necrosis factor-alpha [TNF-α] and chemokines) by infected macrophages or microglia, then activating uninfected macrophages and microglia to produce potentially neurotoxic substances such as quinolinic acid and arachidonic acid [17, 18].

Several regulatory mechanisms for *T. gondii* proteins regulating neuronal apoptosis have been elucidated. *Toxoplasma gondii* granule protein 3 (*Tg*GRA3) activates the neuronal protein kinase R-like endoplasmic reticulum kinase (PERK) pathway, and rhoptry protein 18 (*Tg*ROP18) induces endoplasmic reticulum stress, both of which subsequently trigger cellular apoptosis [19, 20]. Additionally, reactive astrocytes produce nitric oxide radicals (NO·), collectively contributing to neuronal damage [21]. Current therapeutic strategies primarily involve molecules that inhibit the inflammatory signaling or traditional Chinese medicine components like ginsenosides [22, 23]. These agents function as anti-inflammatory modulators by targeting the NLRP3 inflammasome signaling pathway within microglial cells [22]. Furthermore, inhibiting the activation of microglia or astrocytes also mitigates neuronal apoptosis by attenuating CNS inflammation [16]. However, this approach may inadvertently reduce immune pressure on *T. gondii*, potentially leading to the reactivation and release of the parasite from *T. gondii* cysts.

The gut microbiota plays a vital role in the bidirectional communication and maintenance of the integrity of the gut-brain axis, which encompasses neurons, neuroendocrine pathways and neuroimmune interactions [24, 25]. *T. gondii* infection induces significant intestinal inflammation, and the severity of infection correlates closely with alterations in the composition of the host gut microbiota [26]. Among innate immune cells, natural killer (NK) cells, CD4^+^T cells and CD8^+^T cells are critical in combating *T. gondii* infection [27, 28], as their secretion of interferon-gamma (IFN-γ) can effectively inhibit parasite proliferation [29, 30]. However, excessive production of pro-inflammatory cytokines and NO can lead to pathological changes in intestinal tissue, including the degradation of tight junction proteins (TJPs) in IECs, loss of IECs and Paneth cells and disruption of the intestinal barrier, resulting in intestinal leakage [31].

Various therapeutic molecules and dietary supplements have been shown promise in attenuating age-related cognitive decline and depressive-like behavior by modulating the gut microenvironment [32, 33]. Supplements with α-linolenic acid can mitigate intestinal inflammation caused by *T. gondii* infection by alleviating the loss of various probiotics, including *Enterobacteriaceae*, *Proteobacteria*, *Shigella* and *Lactobacillus* [26]. Additionally, regulating the activation and recruitment of microglia through supplementation with short-chain fatty acids (SCFAs), derives from the fermentation of gut microbiota, holds potential for promoting recovery post-experimental stroke [32]. This raises the question of whether supplementation of these beneficial bacteria has the potential to achieve therapeutic effects in the treatment of *T. gondii* infection?

During infection the host immune system also releases cytokines and activates immune cells, which not only target the pathogens but can also impact the composition of the normal gut microbiota [34]. Therefore, understanding the effects of the host immune response and the alterations in gut microbiota resulting from *T. gondii* infection is crucial, as these dysbiosis may subsequently influence immune regulation.

The inflammatory processes involved in combating infections can inadvertently worsen tissue damage through the occurrence of cytokine storms and the formation of immune complexes [35]. Therefore, to evaluate the impact of the host immune response’s strength on gut microbiota balance during infection, we chose vimentin gene knockout mice (*vim−/−* mice) and wild-type (WT) mice as experimental models. Vimentin, an intermediate filament cytoskeletal protein, is not only present in the cytoplasm but also in the nucleus and on the cell membrane [36, 37]. It plays a crucial role in facilitating angiogenesis, maintaining cellular structural integrity and intracellular substance transport and regulating cell signaling pathways [38]. Additionally, vimentin is essential for lymphocyte adhesion and transcellular migration [39]. The absence of vimentin impairs immune cell homing and adhesion, leading to a deficiency in the host’s immune response [40]. Furthermore, vimentin acts as a key regulator of metabolic and functional activities in regulatory T cells (Tregs) [41]. However, elevated levels of vimentin not only contribute to autoimmune diseases like osteoarthritis but also serve as a marker for tumor progression [42]. Vimentin is instrumental in major histocompatibility complex (MHC)-mediated antigen presentation during pathogen infections [43–45].

Therefore, elucidating the impact of *T. gondii* infection on the microbiota and the corresponding immune responses may provide insights into potential strategies for mitigating intestinal and brain damage caused by *T. gondii* infection.

## Methods

### Key resources

The key resources used in this study are reported in table form, as follows:

**Table:** 

Reagent or resource	Source	Identifier
*Antibodies*		
anti-NeuN	Abcam	RRID: AB_10711040 Cat. ab104224
anti-Bax	Abcam	RRID: AB_725631 Cat. ab32503
anti-Caspase-3	Cell Signaling Technology (CST)	RRID: AB_2341188 Cat. A9661
anti-Occludin	Abcam	RRID: AB_2737295 Cat. ab216327
anti-ZO-1	Abcam	RRID: AB_2892660 Cat. ab221547
anti-ki67	Abcam	RRID: AB_443209 Cat. ab15580
anti-Iba1	Abcam	RRID: AB_2636859 Cat. ab178846
anti-GFAP	Proteintech	RRID: AB_2109646 Cat. 16825-1-AP
anti-CD45(PerCP-cy5.5)	BD	RRID: AB_10895563 Cat. 561869
anti-CD3e(bv510)	BioLegend	RRID: AB_2562555 Cat. 100234
anti-NK1.1(PE)	BD	RRID: AB_396674 Cat. 557391
anti-CD19(PE-Cy7)	BD	RRID: AB_394495 Cat. 552854
anti-CD4(APC)	BD	RRID: AB_398528 Cat. 553051
anti-CD8(FITC)	BD	RRID: AB_394568 Cat. 553030
anti-F4/80(PE)	BD	RRID: AB_2687527 Cat. 565410
anti-CD11b(FITC)	BD	RRID: AB_396679 Cat. 557396
anti-CD86(PE-Cy7)	BD	RRID: AB_1727518 Cat. 560582
anti-CD206(AF647)	BD	RRID: AB_2739133 Cat. 565250
*Experimental models: organisms/strains*		
Sv129 mice (strain 129-S2)	Vital River Laboratories	N/A
*Toxoplasma gondii* ME49 strain	Laboratory preserved strain	N/A
*Bacterial strains*		
*Lactobacillus murinus*	ATCC	Cat. 35020
*Lactobacillus gasseri*	ATCC	Cat. 33323
*Chemicals, peptides, and recombinant proteins*		
MRS medium	Solarbio	M8540
Indole-3-lactic acid	Macklin	I849392
CH223191	Selleck	S7711
*Critical commercial assays*		
Mouse IL-10 kit	Multi Sciences	EK210/4
Mouse IFN-γ kit	Multi Sciences	EK280/3
*Oligonucleotides (primers)*		
*Toxoplasma gondii*-B1-F	5ʹ-GGAACTGCATCCGTTCATG-3ʹ	
*Toxoplasma gondii*-B1-R	5ʹ-TCTTTAAAGCGTTCGTGGTC-3ʹ	
*Software and algorithms*		
Prism	GraphPad	https://www.graphpad.com/scientific-software/prism/
ImageJ	Java	https://imagej.en.softonic.com/

*ATCC* American Type Culture Collection, *BD* Becton, Dickinson and Company, *Cat.* catalog,* IFN* interferon,* IL* interleukin,* MRS* DeMan, Rogosa and Sharpe culture* N/A* not available, * RRID* Research Resource Identifier

### Experimental animals, *Toxoplasma gondii* and probiotic strains

Specific pathogen-free (SPF) male sv129-WT and sv129-*vim−/−* mice, aged 8 weeks, were orally infected with four cysts of the ME49 strain of* T. gondii* (ME49 cysts) for the analysis of chronic infection, or with twenty ME49 cysts for survival analysis and the *Lactobacillus* transplantation experiment. Mice that received equivalent volumes of phosphate-buffered saline (PBS) via oral inoculation served as mock-treated controls (UI group).

In the *Lactobacillus* transplantation experiment, *Lactobacillus murinus* (type strain: ATCC 35020) and *Lactobacillus gasseri* (type strain: ATCC 33323) were revived from liquid nitrogen and subsequently resuscitated overnight in DeMan, Rogosa and Sharpe culture medium (MRS medium; Solarbio, Beijing, China) under anaerobic conditions. The activated bacterial solution was then transferred to fresh MRS medium and cultured for an additional 24 h in an anaerobic environment until the bacterial concentration reached 10^9^ colony-forming units (CFU)/ml. The activated bacterial solution was stored at 4 °C, and fresh *Lactobacillus s*uspension were prepared 12 h in advance daily for oral transplantation into the mice. *L. murinus* was administered at a dose of 3 × 10^8^ CFU/ml, and *L. gasseri* was given at a dose of 1 × 10^9^ CFU/ml, both via gavage in 200 μl MRS medium. The initial *Lactobacillus* transplantation was performed 4 h following the oral infection with twenty ME49 cysts. An equivalent quantity of *L. murinus* and *L. gasseri* was administered daily at a fixed time for a duration of 8 days.

Indole-3-lactic acid (ILA; Macklin, CAS no.: 1821-52-9; 10 mg/kg) was orally administered once a day for 8 days, following which the jejunal tissues were collected for testing on the ninth day. The aromatic hydrocarbon receptor (AhR) antagonist, CH223191 (Selleck Chemicals, Houston, TX, USA; cat. no.: S7711; 20 mg/kg), was orally administered every 2 days for a total of 8 days, and peripheral blood samples were collected on the ninth day for flow cytometry detection of CD8^+^ T-cell density.

### Behavioral assessment of mice with *T. gondii* chronic infection

#### Morris water maze test

The Morris water maze test is used to assess spatial learning and memory ability. Mice were acclimated to the experimental environment in the behavioral laboratory 1 week prior to the initiation of the behavioral assessments. The water maze consisted of a cylindrical pool (diameter: 120 cm; height: 60 cm) filled with opaque water containing food-grade titanium dioxide which allowed the mice to be traced and which obstructed the mice’s field of view, preventing them from seeing the submerged platforms. The water depth was maintained at 30 cm, and the temperature was controlled at 21 ± 1 °C.

During the place navigation trial, a hidden platform was placed 1 cm below the water surface in the target quadrant. The mice underwent training for 5 consecutive days, with a maximum time limit of 90 s to locate the platform. Each day, the mice were gently and slowly placed into the water facing the pool wall, each time at a different starting position in a pseudo-random manner. A mouse was considered to have located the platform if it remained on it for at least 5 s. The duration for mice to find the hidden platform were recorded as “escape latency” in seconds. If a mouse failed to find the platform within 90 s, it was manually guided to the platform and allowed to stay there for 30 s. The escape latency and swimming paths of the mice were recorded using a video tracking system.

On the sixth day, the spatial probe test was conducted to assess memory retention. During this trial, the platform was removed, and the mice were allowed to swim freely in the pool for 60 s. The number of times each mouse crossed the previous location of the platform was recorded.

#### Forced swimming test

The forced swimming test is a classic test to evaluate the degree of depression in mice. Mice were introduced into a cylindrical glass cylinder (height: 25 cm; diameter: 10 cm) that was filled with water to a depth of 10 cm. Prior to the test, the mice underwent a 15-min swimming training session. After a 24-h interval, the mice were placed in the tank and allowed to swim for 6 min. During this period, the cumulative immobility time of the mice within the last 4 min was recorded. Immobility was defined as the cessation of active movement, such as floating or minimal limb movements solely to maintain their heads above the water.

#### Novel object recognition test

The novel object recognition test is used to evaluate the memory and cognitive function of mice by comparing the time spent exploring new and old objects. Mice were gently introduced into the central area of a white plastic open-field test box measuring 40 × 40 × 28 cm. Object A and B1, made from the same material but differing in shape, were positioned diagonally within the open-field test box. Upon placing the mice in the center of the open field, the duration of contact with objects A and B1 was recorded. Subsequently, the mice were removed and the open field was cleaned with 75% alcohol to remove any residual odor. One hour later, object B1 was replaced with a new object, B2, and the duration of interactions with objects A and B2 was recorded once again. The recognition index (RI) was calculated using the following formula:

$${\text{RI}} = \left( {{\text{Time spent with B2}} - {\text{Time spent with B1}}} \right)/\left( {{\text{Time spent with A}} + {\text{Time spent with B2}}} \right).$$

#### Elevated plus maze test

The elevated plus maze test is used to evaluate the anxiety of mice by inducing fear of the maze and the impulse to explore at the same time, resulting in the conflict between avoidance and exploration. The mice were placed in the central area of an elevated plus maze, which consisted of four arms—two open arms without walls and two closed arms with walls (length: 25 cm; width: 5 cm; elevation above the floor: 60 cm). The mice were positioned with their heads facing an open arm, and this consistent starting position was maintained for all subsequent trials. The distance traveled by the mice in both the open and closed arms within a 5-min period was recorded. The percentage of distance traveled in the open arm was calculated as the distance traveled in the open arm divided by the total distance traveled in both the open and closed arms.

#### Three-chamber free exploring test

The three-chamber free exploring test is used to evaluate sociability, social novelty and social memory. The three boxes were partitioned by a transparent glass resin plate, and the test mice were placed in the central box for a 5-min acclimation period. Randomly, a novel mouse (stranger 1) was placed in the metal cage on either the left or the right-side chamber, while the metal cage on the opposite side remained empty. Following the removal of the glass resin plate separating the boxes, the test mice were allowed to freely explore all three chambers for 10 min. The duration of direct contact between the test mice and stranger 1 was recorded, with “direct contact” being defined as the test mouse coming approximately 3–5 cm around the metal cage. Subsequently, a second novel mouse (stranger 2) was introduced into the previously vacant metal cage, and the duration of contact between the test mice and stranger 2 was recorded over a period of 10 min.

#### Open field test

The open field test is a classical method to evaluate motor function and anxiety behavior in mice. The mice were gently introduced into the central area of a white plastic open field test box measuring 40 × 40 × 28 cm. They were given 5 min to freely explore the surroundings. A video camera, positioned directly above the arena, was utilized to monitor and record the movement of each mouse. The recorded video data allowed for the measurement of the total distance traveled by each mouse, as well as the distance spent in both the center and periphery of the chamber. The amount of defecation in the open field was also recorded for each mouse. Following each trial session, the arena was thoroughly cleaned using 75% ethanol.

### Detection of parasitic burden and cyst formation in mice with *T. gondii* acute infection and chronic infection, respectively

Sv129-WT and sv129-*vim−/−* mice (*n* = 40 per group) were infected with four ME49 cysts. At 8 days post-infection (dpi) in the *Lactobacillus* transplantation experiment, mice were euthanized and dissected, followed by cardiac perfusion. Tissues, including the jejunum, liver and brain (25 mg each sample), were collected for genomic DNA extraction. The DNeasy Blood and Tissue Kit (Qiagen, Hilden, Germany) was utilized for DNA extraction. *Toxoplasma gondii* burden was quantified by quantifying B1 gene copies in an 1-mg DNA sample using quantitative PCR (qPCR) with specific primers (forward primer: 5ʹ-GGAACTGCATCCGTTCATG-3ʹ; reverse primer: 5ʹ-TCTTTAAAGCGTTCGTGGTC-3ʹ) and Hieff® qPCR SYBR® Green Master Mix (Low Rox Plus; Yeasen, Shanghai, China).

In the cyst formation assay, sv129-WT and sv129-*vim−/−* mice (*n* = 12 per group) at 3 months post-infection (mpi), as well as four groups of sv129-WT mice in the *Lactobacillus* transplantation experiment (*n* = 30 per group) at 30 dpi, were euthanized. Their brains were harvested and homogenized, and 150 μl of homogenate of each mouse was smeared on a slide. The cysts were counted in a double-blind manner under a microscope (Nikon Corporation, Tokyo, Japan).

### RNA sequencing analysis of the mice with *T. gondii* chronic infection

For the RNA sequencing (RNA-seq) experiments, sv129-WT and sv129-*vim−/−* mice (*n* = 3 per group) infected with four ME49 cysts were euthanized at 90 dpi. Total RNA was extracted using TRIzol Reagent (Invitrogen, Thermo Fisher Scientific, Waltham, MA USA) following the manufacturer’s instructions. Subsequently, reverse transcription and library construction were conducted by Majorbio Biopharm Biotechnology (Shanghai, China) in accordance with the manufacturer’s protocol (Illumina Inc., San Diego, CA, USA).

The quality of RNA samples was assessed using a 5300 Bioanalyzer (Agilent Technologies, Santa Clara, CA, USA), and the quantity was measured on the NanoDrop-2000 system (Thermo Fisher Scientific). Only high-quality RNA samples meeting the following criteria were used for library preparation: optical density (OD)260/280 = 1.8–2.2; OD260/230 ≥ 2.0; RNA integrity number (RIN) ≥ 6.5; 28 S:18 ratio ≥ 1.0; > 1 μg of total RNA. The RNA-seq transcriptome library was constructed following the Illumina Stranded mRNA Prep Ligation Reference Guide (Illumina Inc.) using 1 μg of total RNA as previously described [46].

To identify differentially expressed genes (DEGs) between two groups, the level of each transcript was quantified using the transcripts per million reads (TPM) method. The Gene Ontology (GO) database and Kyoto Encyclopedia of Genes and Genomes (KEGG) database were utilized to annotate significantly enriched DEGs that showed significant enrichment with a Benjamini and Hochberg (BH)-corrected false discovery rate (FDR)  < 0.05  (DESeq2, USA). The Reactome database was also used for enrichment analysis, comparing the list of DEGs or differentially expressed proteins to the Reactome pathway database to identify pathways that were significantly enriched.

The data obtained from analyzing KEGG signaling pathways were imported into the Heatmap Dendrogram plug-in of OriginPro (version 2021, 9.8.0; OriginLab, Northampton, MA, USA) to perform heatmap analysis of apoptosis-related genes and toxoplasmosis-related genes. This analysis involved calculations and the drawing of heat maps to visualize the gene expression patterns.

### RNA extraction and quantitative reverse transcription PCR

At 3 mpi, brain tissues were collected from mice with chronic infection. Total RNA was extracted using TRIzol Reagent following the manufacturer’s instructions (Invitrogen,, Thermo Fisher Scientific). Quantitative reverse transcription PCR (qRT-PCR) was performed using HiScript III All-in-one RT SuperMix Perfect for qPCR (Vazyme, Nanjing, China) according to the manufacturer’s protocol. Real-time PCR targeting the *bcl2* gene (forward primer: 5ʹ-GCTACCGTCGTGACTTCGC-3ʹ; reverse primer: 5ʹ-CCCCACCGAACTCAAAGAAGG-3ʹ) and the *bax* gene (forward primer: 5ʹ-AGACAGGGGCCTTTTTGCTAC-3ʹ; reverse primer: 5ʹ-AATTCGCCGGAGACACTCG-3ʹ) was performed on a QuantStudio 6 Real-Time PCR System (Thermo Fisher Scientific) using Hieff® qPCR SYBR Green Master Mix (Low Rox Plus) (Yeasen, Shanghai, China). The specificity of the PCR amplification was confirmed by dissociation curve analysis. Each sample was run in triplicate/quadruplicate, and relative quantitation was determined using the comparative Ct method (2^−ΔΔCT^) with data normalized to the *gapdh* gene. Data were obtained from three independent experiments.

### 16S ribosomal RNA sequencing for fecal samples

For the 16S ribosomal RNA (rRNA) experiments, fecal samples were collected from sv129-WT and sv129-*vim−/−* mice (*n* = 6 per group) infected with four ME49 cysts at 0, 30 and 90 dpi, or from sv129-WT and sv129-*vim−/−* mice (*n* = 6 per group) infected with twenty ME49 cysts at 0, 2 and 7 dpi. The collected samples were stored in liquid nitrogen. For analysis, the microbial DNA was extracted from the fecal samples using the HiPure stool DNA Kit (Magen Biotechnology, Guangzhou, China) following the manufacturer’s instructions. PCR amplification targeting the V3-V4 region of the rRNA gene was performed using the forward primer 5ʹ-CCTACGGGNGGCWGCAG-3ʹ and the reverse primer 5-GGACTACHVGGGTATCTAAT-3ʹ. PCR was carried out using Phanta Max Super-Fidelity DNA Polymerase (Vazyme). The purified amplicons were pooled in equimolar ratios and sequenced using the paired-end strategy (2 × 250) on the Illumina HiSeq 2500 platform following the standard protocols (Illumina Inc.). Bioinformatic analysis was performed using Omicsmart, a dynamic real-time interactive online platform for data analysis (http://www.omicsmart.com).

### Flow cytometry

#### Detection of NK cells, T lymphocytes and B lymphocytes in peripheral blood

Blood samples from sv129-WT mice in four groups (*n* = 6 per group) at 8 dpi were collected into anticoagulation tubes and allowed to stand at 4 °C for 2 h, following which the serum was transferred and stored at − 80 °C. Erythrocytes were removed from the serum by treating the hematocytes with erythrocyte lysis buffer (BD, Franklin Lakes, NJ, USA) following the manufacturer’s instructions. Approximately 1 × 10^6^ cells were counted and washed once with PBS. Fc receptor blocker (CD16/CD32; BD) was added, followed by the addition of detection antibodies, namely anti-CD45-PerCP-cy5.5 (BD), anti-CD3e-bv510 (BD), anti-NK1.1-PE (BD), anti-CD19-PE-Cy7 (BD), anti-CD4-APC (BD) and anti-CD8-FITC (BD).

#### Detection of M1 and M2 macrophages in peritoneal fluid

After the mice were anesthetized and blood samples collected, the mice were euthanized and the abdominal skin was incised to carefully expose the abdominal cavity. Five milliliters of PBS was injected into the abdominal cavity, and the mouse was gently shaken to ensure even distribution of PBS and cells within the cavity. The peritoneal fluid was aspirated and washed once with PBS. The peritoneal fluid containing white blood cells was collected, and Fc receptor blocker (CD16/CD32) was added to prevent nonspecific binding, followed by the addition of detection antibodies, namely anti-CD45-PerCP-cy5.5 (BD), anti-F4/80-PE (BD), anti-CD11b-FITC (BD), anti-CD86-PE-Cy7 (BD) and anti-CD206-AF647 (BD).

### Enzyme-linked immunosorbent assay

Serum was collected from four groups of sv129-WT mice (*n* = 6 per group) at 8 dpi to quantify the concentration of IFN-γ, interleukin (IL)-10 and TNF-α using enzyme-linked immunosorbent assay (ELISA) kits (Multi Sciences) or the concentration of ILA using an ELISA kit from Jiangsu Meimian Industrial Co., Ltd (Jiangsu, China) following the manufacturer’s instructions.

### Histopathological analysis

Jejunum, ileum and brain tissues from the four groups of mice at 8 and 30 dpi were harvested and fixed in 4% paraformaldehyde. The tissues were then embedded in paraffin and sectioned into 4-μm-thick slices. Longitudinal sections were obtained from the small intestine, while coronal sections were obtained from the brain. In both the immunofluorescence and the hematoxylin and eosin (HE) staining procedures, the paraffin sections were subjected to deparaffinization, dehydration and rehydration steps. In the HE staining procedure, eosin stains the cytoplasm and extracellular matrix and hematoxylin stains nuclei.

The histological score (HE scores), assessed using a 6-point scale, was determined for the jejunum and ileum tissues. This evaluation was performed by analyzing five high-power fields of view for each intestinal slice, following the reference method [47, 48]. To measure the length of the villi and depth of the crypts of the jejunum and ileum, one low magnification field of view was randomly selected for each intestinal slice. The length of the 10 longest villi and the depth of the 10 deepest crypts were measured, resulting in a total of 60 data points per tissue type. The HE scores and length measurements were independently performed by two individuals.

### Immunofluorescence analysis

After dewaxing and hydrating, the paraffin sections underwent heat-mediated antigen retrieval using Tris/EDTA buffer (pH = 9.0) or citrate buffer (pH = 6), following the instructions provided by the manufacturers of the respective antibodies. The sections were then permeabilized with 0.2% Triton-X100 for 10 min and blocked with 5% donkey serum for 1 h at room temperature, following which the sections were incubated overnight at 4 °C with the primary antibody, including anti-NeuN (1:400; ab104224; Abcam, Cambridge UK), anti-NeuN (1:200; 26975-1-AP; Proteintech, Wuhan East Lake High-tech Development Zone, China), anti-Bax (1:100; ab32503; Abcam), anti-Caspase-3 (1:200; 9661; Cell Signaling Technology, Danvers, MA, USA), anti-occludin (1:200; ab216327; Abcam), anti-ZO-1 (1:100; ab221547; Abcam), anti-ki67 (1:100; ab15580; Abcam), anti-GFAP (1:200; 16825-1-AP; Proteintech) and anti-IBA1 (1:2000; ab178846; Abcam). Afterward, the sections were incubated with the secondary antibody (1:1000) for 1 h at room temperature, including goat anti-rabbit (Alexa Fluor 488, 65-6120, or AF594, A11037; Thermo Fisher Scientific), goat anti-mouse (Alexa Fluor 594, A11005, or AF488, A11029), donkey anti-rat (Alexa Fluor 594, A48271). Finally, the sections were sealed with a fluorescent mounting oil containing DAPI (SouthernBiotech, Birmingham, AL, USA).

For the TUNEL (terminal deoxynucleotidyl transferase dUTP nick end labeling) assays, the paraffin sections of the brain were permeabilized with 20 μg/ml Proteinase K for 10 min at room temperature. The TUNEL FITC Apoptosis Detection Kit (Vazyme) was used following the manufacturer’s protocol.

For statistical analysis, five different high-power fields of views were captured from each section. Mean optical density (integral optical density/area) was used to evaluate the expression levels, or TUNEL-FITC-positive/Caspase-3 positive cell counting was conducted. The analysis process was performed using ImageJ software (v1.8.0).

### Statistical analysis

Analyses and graphical representations were performed using SPSS version 20 software (SPSS IBM Corp., Armonk, NY, USA) and GraphPad Prism 8 (GraphPad Software, San Diego, CA, USA). All data were presented as the mean ± standard error of the mean (SEM). Significant differences between groups were assessed using unpaired two-tailed Student’s t-test or one-way analysis of variance (ANOVA), as indicated in the figure legends. Cumulative mortality was depicted using Kaplan–Meier survival plots and analyzed using the Mantel-Cox log-rank test. For HE staining scoring, a two-independent samples Mann–Whitney nonparametric test was utilized. The statistical methods employed for analyzing animal behavior are described in figure legends. Statistical significance was defined as a *P* value < 0.05.

## Results

### Behavioral and brain transcriptome results indicated that WT mice exhibited more severe brain damage in *T. gondii* chronic infection

**Fig. 1:**
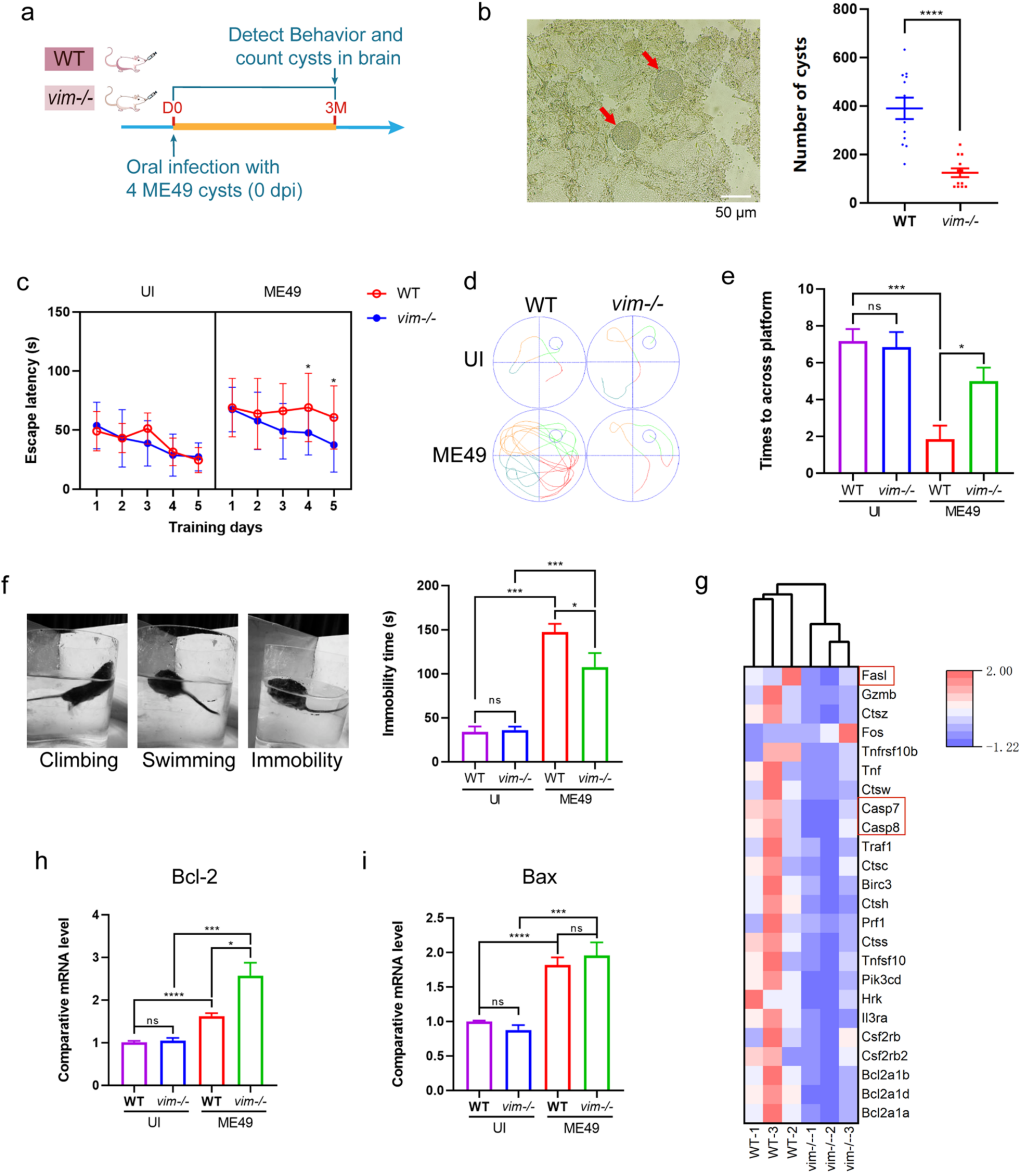
Detection of cognitive impairment caused by chronic *Toxoplasma gondii* infection in two types of mice through behavioral and transcriptome sequencing. **a** Schematic of the experimental design. Eight-week-old male mice strains sv129-WT and sv129-*vim−/−* were orally infected with four ME49 cysts (experimental group) or with an equivalent amount of PBS solution as the uninoculated control, and behavioral testing, brain cyst counts and brain transcriptome analysis were conducted at 3 mpi. **b** Detection and counting of the cyst formation in brain homogenate at 90 dpi under a light microscopy: the representative view for the ME49 cysts and the counting of ME49 cysts in the whole brain. **c**–**e** Behavioral testing of WT and *vim−/−* mice in both uninfected conditions (UI group) and ME49 chronic infection conditions (ME49 group): Morris water maze test showing escape latency (seconds) in the place navigation trial for the first 5 consecutive days (**c**) and the representative actual trajectory on the 5th training day (**d**); times to cross platform in the spatial probe trial (**e**). **f** Forced swimming test: 3 representative tests of tested mice in the water and the cumulative immobility time was recorded. **g** Heatmap of enriched apoptotic genes in KEGG signaling pathway analysis for the DEGs. **h**, **i** Comparative mRNA level of *bcl-2* (**h**) and *Bax* (**i**) in the brain of chronically infected WT and* vim*−/− mice. Means were compared using the unpaired t-test (**b**), or repeated measures two-way ANOVA with Bonferroni post hoc comparison (**c**), or one-way ANOVA with Tukey’s multiple comparison test (**e**, **f**). Asterisks indicate significant differences at *P < 0.05, ***P < 0.001, ****P < 0.0001; ns, no significance. ANOVA, Analysis of variance; Bax, *bcl2*-associated X-protein; Bcl-2, apoptosis inhibiting protein; DEGs, differentially expressed genes; dpi, days post-inoculation; KEGG, Kyoto Encyclopedia of Genes and Genomes; mpi, months post-inoculation; ME49, cysts of the ME-49 strain of* T. gondii*; mRNA, messenger RNA; PBS, phosphate-buffered saline; UI, uninfected; *vim−/−*, vimentin gene knockout mice; WT, wild-type mice

To elucidate the distinctions in neuropsychiatric disorders between sv129-WT mice and sv129-*vim−/−* mice caused by chronic *T. gondii* infection, we conducted a series of behavioral tests at 3 months post-infection (mpi, ME49 group), with uninfected mice as controls (UI group) (Fig. 1a). Firstly, we found a significant increase in cyst formation in WT mice compared to *vim−/−* mice (Fig. 1b). The result of the Morris water maze test revealed a significant decrease in escape latency in the place navigation trial in UI and *vim−/−* ME49 group (Fig. 1c, d and Fig. S1a), indicating a decrease in learning ability in the WT-ME49 group. In the spatial probe trial, WT-ME49 mice spent the longest distances searching for platforms, and the times of crossing platforms significantly decreased (Fig. 1e), suggesting the greatest decline in learning and spatial memory abilities in the infected WT mice.

The forced swimming test was used to measure depression-like symptoms by identifying three behavioral states, namely climbing, swimming and immobility, with the duration of the immobility state recorded (Fig. 1f). The results demonstrated that the immobility time recorded in the ME49 groups was significantly longer than that in the UI groups, and that the immobility time in WT ME49 mice was significantly longer than that in *vim−/−* ME49 mice (Fig. 1f). This result indicates that *T. gondii* infection significantly induced depression-like symptoms in both WT and *vim−/−* mice, with infected WT mice presenting more severe impairment than *vim−/−* mice.

The novel object recognition test revealed that *T. gondii* infection significantly impaired the mice’s memory (Additional file 1: Figure S1b), which is consistent with our findings in the Morris water maze test (Fig. 1c–e). In the elevated plus maze test, there was a significant reduction in anxiety levels among *T. gondii*-infected mice (Additional file 1: Figure S1c), further supported by the diminished social novelty observed in the three-chamber free exploring test (Additional file 1: Figure S1d). Notably, vimentin deficiency may impair the social abilities of the mice (Additional file 1: Figure S1e). The open field test indicated a decrease in the amount of feces in *T. gondii*-infected mice (Additional file 1: Figure S1f), indicating a reduction in anxiety levels that observed in the elevated plus maze test (Additional file 1: Figure S1c). Although no significant differences were observed in the percentage of center distance in the open field test, this could be attributed to individual variations among the mice (Additional file 1: Figure S1g).

Transcriptome sequencing was conducted to examine the differences in gene transcription levels in the brains of the two mouse strains in the *T. gondii* chronic infection condition.

The KEGG pathway enrichment analysis revealed notable disparities between WT and *vim−/−* mice with chronic infection, particularly in terms of immune regulation and inflammation-related pathways, such as cell adhesion molecules, hematopoietic cell lineage, antigen processing and presentation, cytokine-cytokine receptor interaction and apoptosis (Additional file 1: Figure S1h). Notably, the KEGG analysis identified significant differences in apoptosis-related genes (Fig. 1g). Specifically, genes associated with both exogenous and endogenous apoptosis, including *fasl* (Fas ligand), *casp7* (caspase-7), *casp8* (caspase-8), and *hrk* (harakiri, BCL2 interacting protein), were generally up-regulated in WT mice with chronic infection (Fig. 1g).

We further observed a significant increase in the transcription levels of *bax* (*bcl2*-associated X-protein, an endogenous apoptotic marker) and *bcl2* (the apoptosis inhibiting gene) in the brain compared to the UI group. Importantly, the transcription level of *bcl2* was comparatively lower in WT mice compared to *vim−/−* mice, while no significant difference in *bax* transcription was observed between the two mouse strains (Fig. 1h, i).

Additionally, the enrichment of genes related to toxoplasmosis from KEGG analysis indicated variability in disease severity between the two mouse strains (Additional file 1: Figure S1i).

These findings suggest that, compared to *vim−/−* mice, WT mice exhibited a higher rate of cyst formation and experienced more pronounced neurological and psychiatric abnormalities following infection. Based on these findings, we hypothesize that these outcomes could be attributed to the robust immune response observed in WT mice, potentially involving the induction of cytokine storms during the anti-infection process, which in turn can result in tissue barrier damage.

### WT mice experienced more severe neuronal apoptosis in the brain during *T. gondii* chronic infection

**Fig. 2:**
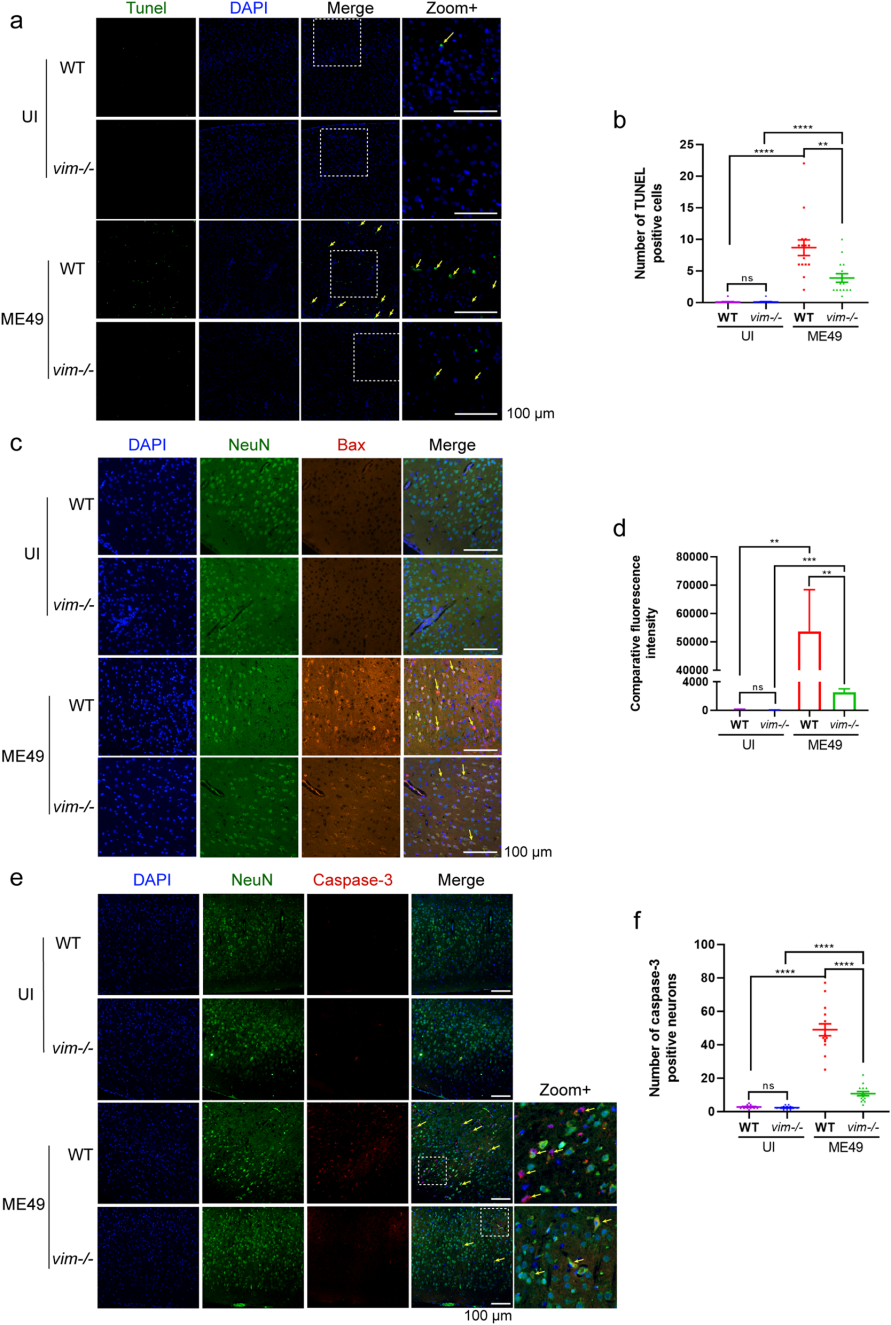
Detection of differences in neuron apoptosis between two types of mice with chronic *T. gondii* infection. **a**, **b** Detection of cell apoptosis with TUNEL staining (*n* = 6): representative immunofluorescence images (**a**) and quantification of TUNEL-FITC positive-stained cells (**b**). **c**, **d** Detection of neuron apoptosis using the neuron marker NeuN and endogenous apoptosis marker Bax (**c**), and quantification of fluorescence intensity of Bax (**d**) (*n* = 6). **e**,** f** Detection of neuron apoptosis using the neuron marker NeuN and cell apoptotic marker caspase-3 (**e**), and quantification of caspase-3 positive neurons (**f**) (*n* = 6). Arrows indicate positive staining. The fluorescence intensity and positive counting were measured in randomly selected high-power fields. Scale bar: 100 µm. Statistical significance was determined using one-way ANOVA. Asterisks indicate significant differences at **P < 0.01, ***P < 0.001, ****P < 0.0001; ns, no significance. ANOVA, Analysis of variance; Bax, *bcl2*-associated X-protein; DAPI, fluorescent stain; FITC, fluorescein isothiocyanate; ME49, cysts of the ME49 strain of* T. gondii*; TUNEL, terminal deoxynucleotidyl transferase dUTP nick end labeling; UI, uninfected; *vim−/−*, vimentin gene knockout mice; WT, wild-type mice

To elucidate the specific differences in CNS neuronal apoptosis during *T. gondii* chronic infection between WT and *vim−/−* mice, we utilized TUNEL staining to detect apoptosis in situ. The results revealed a higher incidence of positive FITC (fluorescein isothiocyanate)-labeling in WT mice post-infection (Fig. 2a, b). Immunofluorescence analysis of Bax and the neuronal marker NeuN demonstrated a more pronounced neuronal apoptosis in WT mice post-infection (Fig. 2c, d), leading to an upregulated expression of caspase-3 (Fig. 2e, f).

These results suggest that increased neuronal apoptosis may contribute to the heightened depressive tendencies observed in WT mice with chronic infection. Consequently, we asked the questions: What is the relationship between the gut microbiota and the more severe brain injury observed in WT mice? What are the distinctive characteristics of gut microbiota changes during infection in mice with different immune states? How does this increased neuronal apoptosis relate to the more severe disease course observed in WT mice?

### *Toxoplasma gondii* infection resulted in sustained disruption of gut microbiota composition and intestinal inflammation in WT mice

**Fig. 3:**
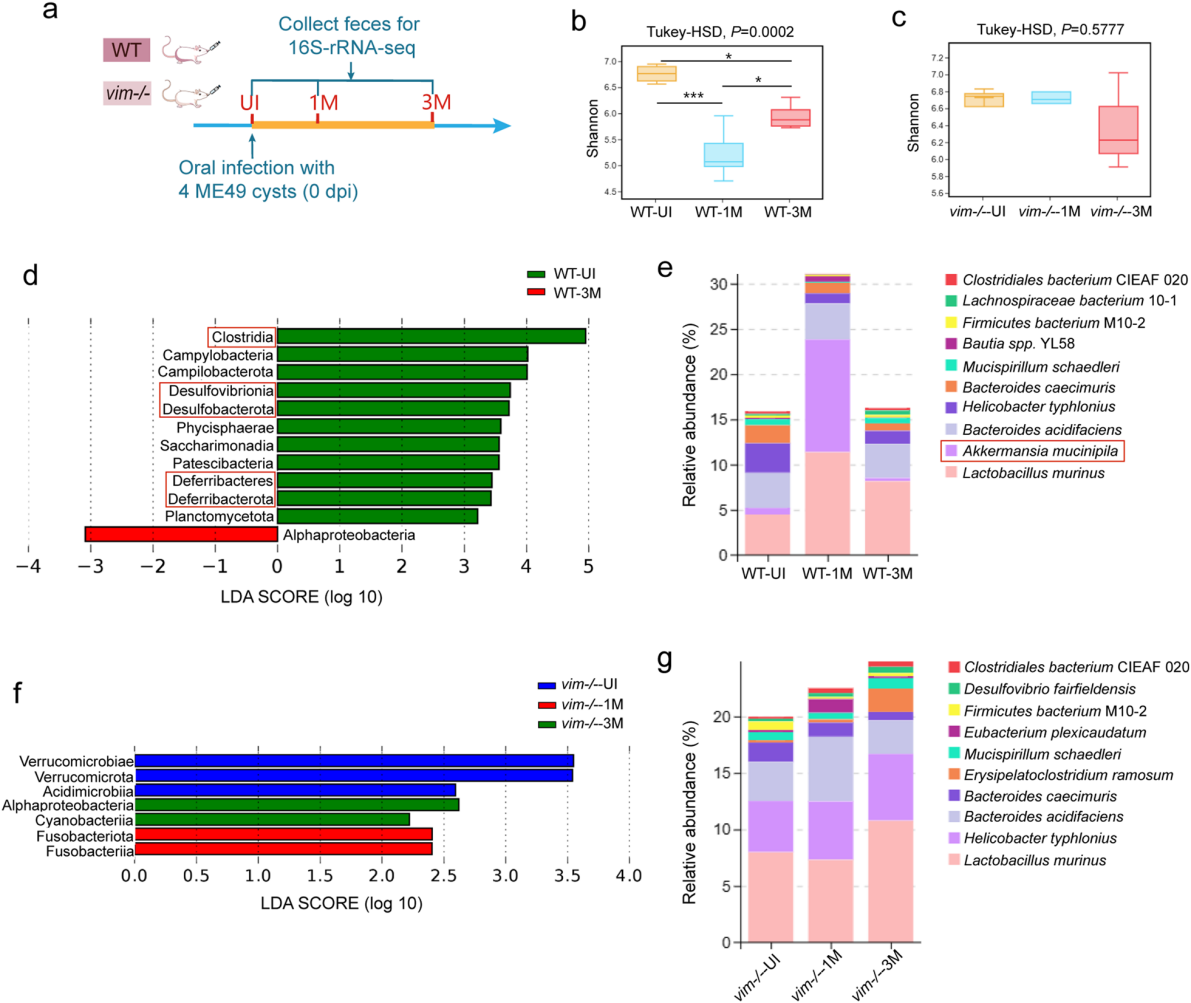
Detection of differences in gut microbiota changes between two types of mice during chronic *T. gondii* infection. **a** Schematic of the experimental design. Feces of WT and *vim−/−* mice were collected for 16S rRNA sequencing before infection (UI group), at 1 mpi (1M group) and at 3 mpi (3M group), respectively (*n* = 6). **b**, **c** Shannon index was used in alpha-analysis at the species level to compared the microbiota diversity among the three time points of WT (**b**) and *vim−/− *(**c**) mice. **d**, **f** Histogram of the LDA score in LEfSe analysis showing the dominant bacteria in the gut microbiome at three time points in WT (**d**) and *vim−/−* (**f**) mice; species with LDA score (log 10) > 2 are shown. **e**, **g** Relative bacterial abundance at the species level of WT (**e**) and *vim−/−* (**g**) mice. Results are expressed as the mean ± SEM and determined by Turkey-HSD test in (**b**, **c**). Asterisks indicate significant differences at *P < 0.05, *** P < 0.001. LDA, Linear discriminant analysis score; LEfSe, LDA effect Size; ME49, cysts of the ME49 strain of* T. gondii*; mpi, months post-infection; rRNA, ribosomal RNA; SEM, standard deviation of the mean; UI, uninfected; *vim−/−*, vimentin gene knockout mice; WT, wild-type mice

Firstly, to evaluate the impact on the gut microbiota composition in the two groups of mice during *T. gondii* chronic infection, we collected fecal samples at 0 (pre-infection), 1 mpi (1M group) and 3 mpi (3M group) for 16S rRNA sequencing (Fig. 3a). Alpha-diversity analysis of the microbial communities was conducted using the Shannon index, which measures both richness and evenness (Fig. 3b, c). In WT mice, a significant decrease in species richness was observed after infection, with the abundance of the microbial community at 1 mpi being lower than at 3 mpi, indicating a decrease in microbial diversity followed by a subsequent increase during infection (Fig. 3b). However, in *vim−/−* mice, no significant changes were detected in the bacterial community abundance throughout the study period (Fig. 3c).

To identify differential microbial taxa between these groups, we employed linear discriminant analysis effect size (LEfSe) (Fig. 3d, f), which combines statistical tests and linear discriminant analysis (LDA) to identify features with significant differences in abundance between groups and assigns them a LDA score plotted on the* x*-axis. In both the WT-1M and WT-3M groups, a significant decrease was observed in the abundance of several phyla, including *Firmicutes*, *Desulfobacterota*, *Bacteroidota* and *Deferribacteres* (Fig. 3d). This reduction pattern is consistent with gut microbiota alterations observed in inflammatory bowel disease (IBD), such as Crohn’s disease [49]. Notably, the most significant reduction in species abundance was observed in the WT-1M group (Fig. 3d). Additionally, a notable increase in the abundance of *Akkermansia muciniphila* was observed in the WT-1M group, followed by a decline in the WT-3M group (Fig. 3e). *Akkermansia muciniphila* has been strongly linked to the improvement of intestinal inflammation and has beneficial effects on various metabolic diseases [50, 51].

Conversely, no significant loss in the abundance of dominant bacterial taxa and the upregulation of *A. muciniphila* were detected in *vim−/−* mice after infection across the three time points (Fig. 3f, g). These findings suggested that chronically infected WT mice exhibited a more severe dysbiosis of the gut microbiota, characterized by loss of species diversity and absence of indicator species, along with heightened intestinal inflammation compared to *vim−/−* mice within 30 dpi.

### The intestinal colonization period of *T. gondii* led to a decrease in the abundance of various *Lactobacilli*

**Fig. 4:**
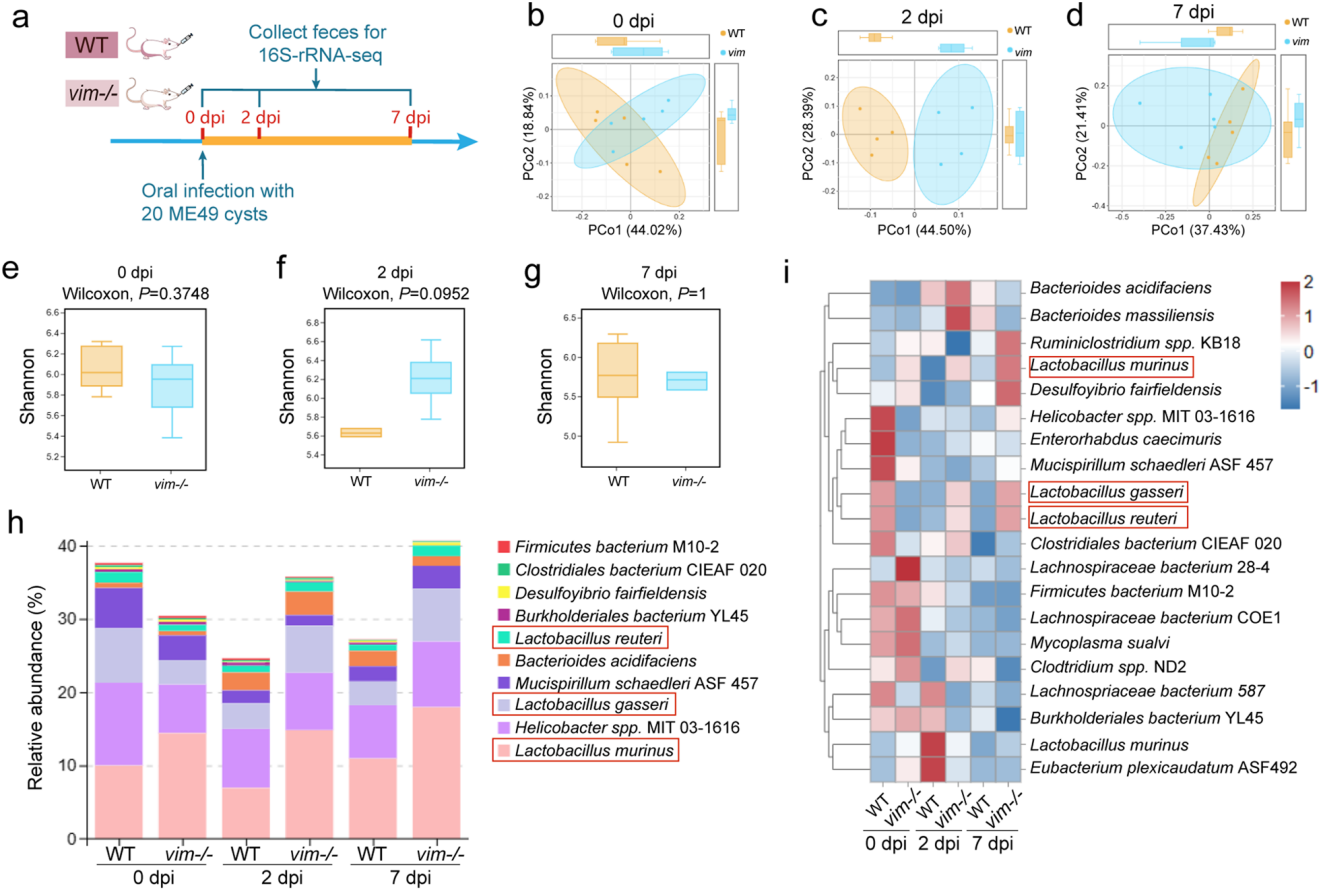
Detection of the effects of intestinal inflammation on microbial composition and abundance in WT mice during infection. **a** Schematic of the experimental design. Feces of WT and *vim−/−* mice were collected for 16S rRNA sequencing before infection (0 dpi), at 2 dpi and at 7 dpi, respectively (*n* = 6). **b-d** PCoA analyses were used in beta-analysis based on the weighted UniFrac Distances of OTUs between two groups of mice at 0 (**b**), 2 (**c**) and 7 (**d**) dpi. **e**–**g** Shannon index was used in alpha-analyses at the species level between two groups of mice at 0 (**e**), 2 (**f**) and 7 (**g**) dpi. **h** Relative bacterial abundance at the species level between WT and *vim−/−* mice at three time points. **i** The species clustering heatmap showed the relative changes in the species proportion between WT and *vim−/−* mice at three time points (with species tags/total tags > 0.1% were shown). dpi, Days post-infection; ME49, cysts of the ME49, strain of* T. gondii*; OTU, operational taxonomic unit; PCoA, principal coordinates analysis; *vim−/−*, vimentin gene knockout mice; WT, wild-type mice

To investigate the specific differences in gut microbiota between WT and *vim−/−* mice within 30 dpi, fecal samples were collected for 16S rRNA sequencing at three time points: before infection (0 dpi), 2 dpi (when *T. gondii* had invaded or started proliferating in IECs) and 7 dpi (when *T. gondii* had extensively proliferated in or had destroyed IECs) (Fig. 4a). Beta-analysis revealed a significant disparity in microbial community composition between these two mouse groups at 2 dpi (Fig. 4c), while no distinct separation was observed at 0 dpi and 7 dpi (Fig. 4b, d). Alpha-analysis indicated no significant differences in species abundance between the two mouse groups at these three time points, although there was a downward trend in the WT group at 2 dpi (Fig. 4e–g).

To identify specific bacterial genera that changed, we conducted a species-level compositional analysis (Fig. 4h, i). The species stacking diagram revealed a significant reduction in the proportion of various lactobacilli, including *L. murinus*, *L. gasseri* and *L. reuteri*, in WT mice after infection (Fig. 4h). Notably, *L. murinus* constituted the largest proportion, while *L. gasseri* exhibited the most significant reduction, followed by *L. reuteri* (Fig. 4h). The heatmap was used for species composition analysis, and revealed consistently higher proportions of *L. murinus* in *vim−/−* mice compared to WT mice (Fig. 4i). The proportions of *L. gasseri* and *L. reuteri* in the WT group not only decreased post-infection, but also showed a reversal trend, with higher proportions observed in the *vim−/−* group (Fig. 4i). It is noteworthy that, when excluding the *vim−/−* group and examining the trend of changes in WT mice separately, the trend of changes in these *Lactobacillus *spp. remains consistent, that is, their proportion decreases after infection. This indicates that utilizing immunosuppressive mouse models other than *vim−/−* mice would likely yield similar results in terms of the observed trend.

### *Lactobacillus murinus* and *L. gasseri* transplantation significantly alleviated small intestinal lesions caused by *T. gondii* infection

Probiotic lactobacilli, which are integral members of the natural microbiota, can rectify ecological imbalances and bolster microbial community resilience [52, 53]. Lactobacilli can produce SCFAs, amines, indoles and other small metabolites, thus playing a protective role in the mucosal immune system against pathogens and mitigating microbial dysbiosis [54, 55].* Lactobacillus murinus* has been shown to promote the release of IL-10 from M2-macrophages through the Toll-like receptor 2 (TLR-2) signaling pathway, thereby alleviating intestinal ischemia–reperfusion injury [56]. *Lactobacillus gasseri* has been found to alleviate insulin resistance and liver injury induced by type 2 diabetes through the intestinal hepatic axis, possibly due to its antibacterial activity, bacteriocin production and immunomodulatory effects on both innate and adaptive systems [53]. Given these findings, it is worth considering whether the abundance of these two lactobacilli is related the severity of *T. gondii* infection in WT mice.

**Fig. 5:**
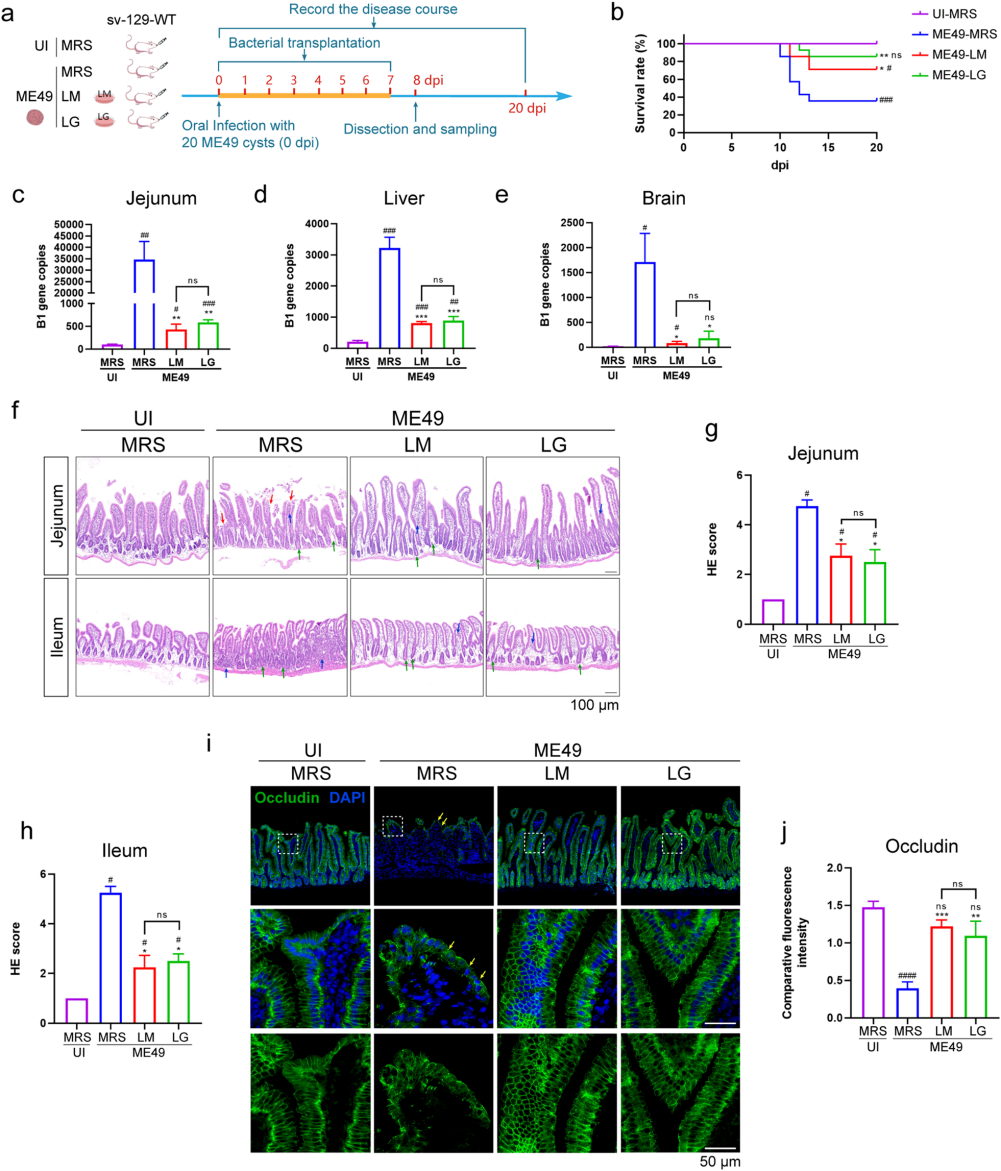
Detection of the regulation of host immune response during *T. gondii* infection after transplantation of two *Lactobacillus* strains. **a** Schematic of the experimental design. Eight-week-old sv129-WT mice were randomly divided into four groups and were oral infected with 20 ME49 cysts (ME49 group) or an equivalent volume of PBS solution (UI group). Four hours post-infection, *Lactobacillus murinus* (LM) and *Lactobacillus gasseri* (LG) were garaged separately at the fixed time every day for 8 days, and MRS medium was fed as control (MRS group). **b** The survival rates of the four groups were recorded for 20 days. **c**–**e** Mice were dissected at 8 dpi for *T. gondii* burden detection in the tissue of the jejunum (**c**), liver (**d**) and brain (**e**). **f** HE staining shows various degrees of damage in the jejunum and ileum in the UI-MRS, ME49-MRS, ME49-LM and ME49-LG groups at 8 dpi (*n* = 6). Red arrows indicate villi rupture or exposure of the intestinal lamina propria, blue arrows indicate inflammatory cell infiltration and green arrows indicate small intestine crypts. Scale bar: 100 μm. **g**, **h** HE scores were used to evaluate the pathological rating of jejunum (**g**) and ileum (**h**) based on the HE staining. **i**, **j** Representative immunofluorescence images (**i**) and quantification (**j**) of fluorescence intensity of occludin. Scale bar: 50 μm. Statistical significance was determined using the two independent samples Mann–Whitney test in HE scores analysis (**g**, **h**), or one-way ANOVA with Tukey’s multiple comparison test (**c**–**e**, **j**). The hashtags (#) indicate significant differences in comparison with the UI-MRS group, and the asterisks (*) indicate significant differences in comparison with the ME49-MRS group: #/*P < 0.05, ##/**P < 0.01, ###/***P < 0.001, ####/****P < 0.0001; ns, not significant. ANOVA, Analysis of variance; dpi, days post-inoculation; HE, hematoxylin-eosin stain; ME49, cysts of the ME49 strain of* T. gondii*; MRS, DeMan, Rogosa and Sharpe culture medium; UI, uninfected; *vim−/−*, vimentin gene knockout mice; WT, wild-type mice

*Lactobacillus murinus* and *L. gasseri* were individually cultured in MRS medium under anaerobic conditions for functional validation. Starting from the day of infection, *L. murinus* and *L. gasseri* transplantation was performed via gavage every 24 h for 8 consecutive days, denoted as the ME49-LM and ME49-LG group, respectively (Fig. 5a). Both the uninfected group and the untreated group were given an equal volume MRS medium by gavage, denoted as the UI-MRS and ME49-MRS group, respectively (Fig. 5a). Mice from each group were randomly selected for survival analysis or were euthanized for sample collection (Fig. 5a).

Survival analysis revealed that the transplantation of these two *Lactobacillus* strains significantly improved survival rate of infected mice (Fig. 5b). Transplantation of *L. murinus* and *L. gasseri* increased the survival rates to 71.4% and 85.7%, respectively, whereas the survival rate was only 35.7% in ME49-MRS group (Fig. 5b). The copy number of the *T. gondii* B1 gene was used to evaluate the proliferation of *T. gondii* in the jejunum (the primary colonization site of *T. gondii*), as well as in the liver and brain (to detect the spread of *T. gondii*) (Fig. 5c–e). The results indicated that among these three tissues, *T. gondii* burden was highest in the jejunum, followed by the liver, and least in the brain (Fig. 5c–e). The highest parasite burden in these tissues was in the ME49-MRS group, indicating that the transplantation of *L. murinus* and *L. gasseri* significantly inhibited the proliferation of *T. gondii*.

The HE staining and HE pathological scoring results in both the jejunum and ileum indicated that the ME49-MRS group exhibited more severe lesions compared to the ME49-LM and ME49-LG groups (Fig. 5f–h), manifesting as exposure of the intestinal lamina propria, rupture or abnormal morphology of intestinal villi, and severe infiltration of inflammatory cells. By measuring the villus length of the jejunum and ileum, we found that the villus in the ME49-MRS group was significantly shortened, whereas the ME49-LM and ME49-LG groups did not differ significantly from the UI-MRS group (Additional file 2: Figure S2a, b). The depth of crypts was most significantly deepened in the ME49-MRS group (Additional file 2: Figure S2c, d), while the ME49-LM and ME49-LG groups exhibited an increased trend compared to the UI-MRS group, but exhibited relief compared to the ME49-MRS group (Additional file 2: Figure S2c, d).

To investigate the impact on the intestinal epithelial barrier, we detected the expression of the TJPs occludin and ZO-1 (Fig. 5i, j; Additional figure 2: S2e, f). The results revealed significant reductions in the expression of both occludin and ZO-1 in the ME49-MRS group, while the ME49-LM and ME49-LG groups exhibited significant improvement (Fig. 5i, j; Additional file 2: S2e, f).

In addition to the small intestine, we also measured the colon length as an indicator of colon swelling. The results indicated no significant evidence of colitis, regardless of *T. gondii* infection or *Lactobacillus* administration at 8 dpi (Additional file 2: Figure S2g, h).

Taken together, these findings suggested that the transplantation of *L. murinus* and *L. gasseri* can significantly alleviate small intestinal injury and prevent the disruption of TJPs in IECs caused by *T. gondii*.

### The transplantation of two *Lactobacillus* strains significantly promotes the recovery of *T. gondii* enteritis and reduces brain tissue damage

**Fig. 6:**
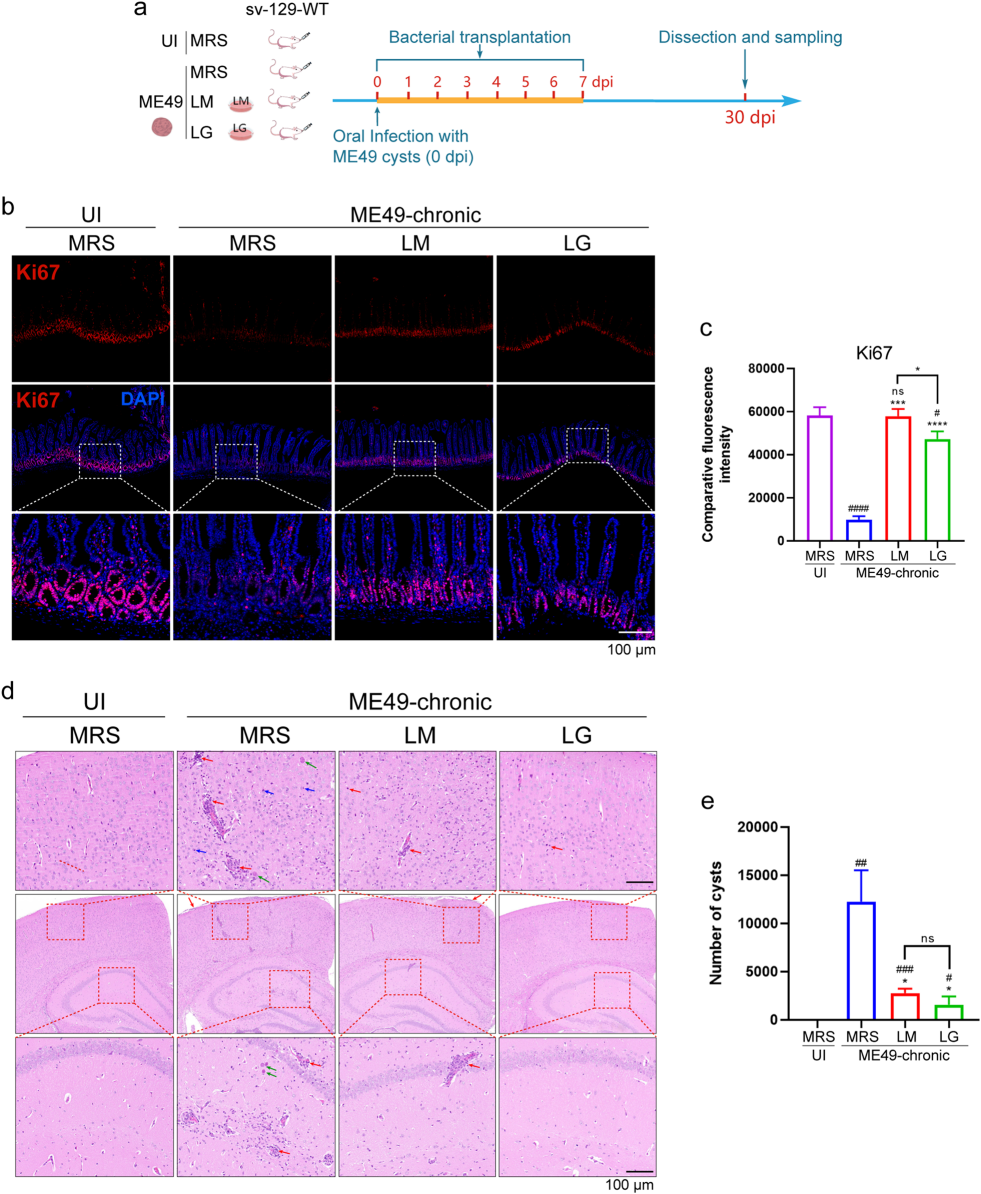
Detection of the effect of transplantation of two *Lactobacillus* strains (LG and LM) on the repair of intestinal injury and CNS inflammation during chronic infection. **a** Schematic of the experimental design. **b**–**c** Representative immunofluorescence images (**b**) and quantification of fluorescence intensity (**c**) of Ki67 in jejunum at 30 dpi. Scale bar: 100 μm. **d** Representative HE staining shows the CNS damage caused by *T. gondii* infection after *Lactobacillus* transplantation at 30 dpi. Green arrows indicate cysts formation, red arrows indicate inflammatory cell infiltration, and blue arrows indicate activated microglia in the prefrontal cortex and hippocampus. Scale bar: 200 μm. **e** ME49 cysts formation in brain was counted. Statistical significance was determined using one-way ANOVA with Tukey’s multiple comparison test (**c**, **e**). The hashtags (#) indicate significant differences in comparison with the UI-MRS group, and the asterisks (*) indicate significant differences in comparison with the ME49-MRS group: #/*P < 0.05, ##/**P < 0.01, ###/***P < 0.001, ####/****P < 0.0001; ns, not significant. ANOVA, Analysis of variance; CNS, central nervous system; LG, *Lactobacillus gasseri*; LM, *Lactobacillus murinus*; ME49, cysts of the ME49 strain of* T. gondii*; MRS, DeMan, Rogosa and Sharpe culture medium; UI, uninfected; *vim−/−*, vimentin gene knockout mice; WT, wild-type mice

At approximately 20 dpi, we observed a gradual return to normalcy in the three infected groups of mice, characterized by the disappearance of symptoms such as ruffled fur and anorexia. However, as evident from the preceding gut microbiota sequencing results (Fig. 3), infection with *T. gondii* resulted in a persistent reduction in the abundance of the microbiota in the mice during this period. Therefore, to determine whether *Lactobacillus* transplantation can migrate intestinal inflammation caused by infection, we examined the repair of the small intestine at 30 dpi (Fig. 6a). The results indicated that the morphology in both the jejunum and ileum in mice with *Lactobacillus* transplantation had largely returned to normal, with residual lesions observed only in the jejunum of the ME49-MRS group (Additional file 3: Figure S3a–c). The length of jejunal villi and depth of the crypts in these groups had also returned to normal levels (Additional file 3: Figure S3d–g). However, the jejunal villi in the ME49-MRS group remained slightly shorter, and the jejunal crypts were deeper, suggesting more persistent damage at these sites (Additional file 3: Figure S3e, f).

The enteritis-induced mucosal damage impairs cell proliferation within the crypt region, thereby hindering tissue repair processes. Consequently, the proliferative capacity of mucosal cells was assessed by examining the expression levels of Ki67 in both small IECs and crypt cells (Fig. 6b, c). The result demonstrated a significantly lower expression level of Ki67 in the non-transplanted group compared to uninfected and *Lactobacillus*-transplanted mice (Fig. 6b, c). This observation supported that the ME49-MRS group experienced a prolonged period of mucosal injury repair, suggesting that their intestinal stem cell regeneration capacity was impeded, resulting in diminished tissue repair ability (Fig. 6b, c).

*Toxoplasma gondii* can cross the BBB and colonize the brain, typically occurring around 7–10 dpi, although the exact timing may vary depending on the *T. gondii* strain; this is followed by the infiltration of inflammatory cells and activation of glial cells within the CNS [6, 57]. We detected the formation of *T. gondii* cysts at 15 dpi, with the cysts gradually thickening their walls and adopting a round shape over time. Therefore, we investigated CNS inflammation at 30 dpi when the infected mice presented psychiatric disorders [58].

HE staining showed high-density cyst formation in the mice of the ME49-MRS group, accompanied by inflammatory cell infiltration and microglial phagocytosis of neurons in both the cortical and hippocampal regions (Fig. 6d). Moreover, upon examination of the entire mouse brain, the ME49-MRS group exhibited the highest cyst count (Fig. 6e). Additionally, immunofluorescence staining of the microglia marker Iba1 and the astrocyte marker GFAP in both the cortical and hippocampal regions demonstrated a significant increase in both the size and number of microglial cells and astrocytes in the ME49-MRS group compared to the *L. murinus*- and *L. gasseri*- treated group (Additional file 4: Figure S4a–f).

### *Lactobacillus murinus* and *L. gasseri* transplantation attenuated neuronal apoptosis induced by *T. gondii* infection

**Fig. 7:**
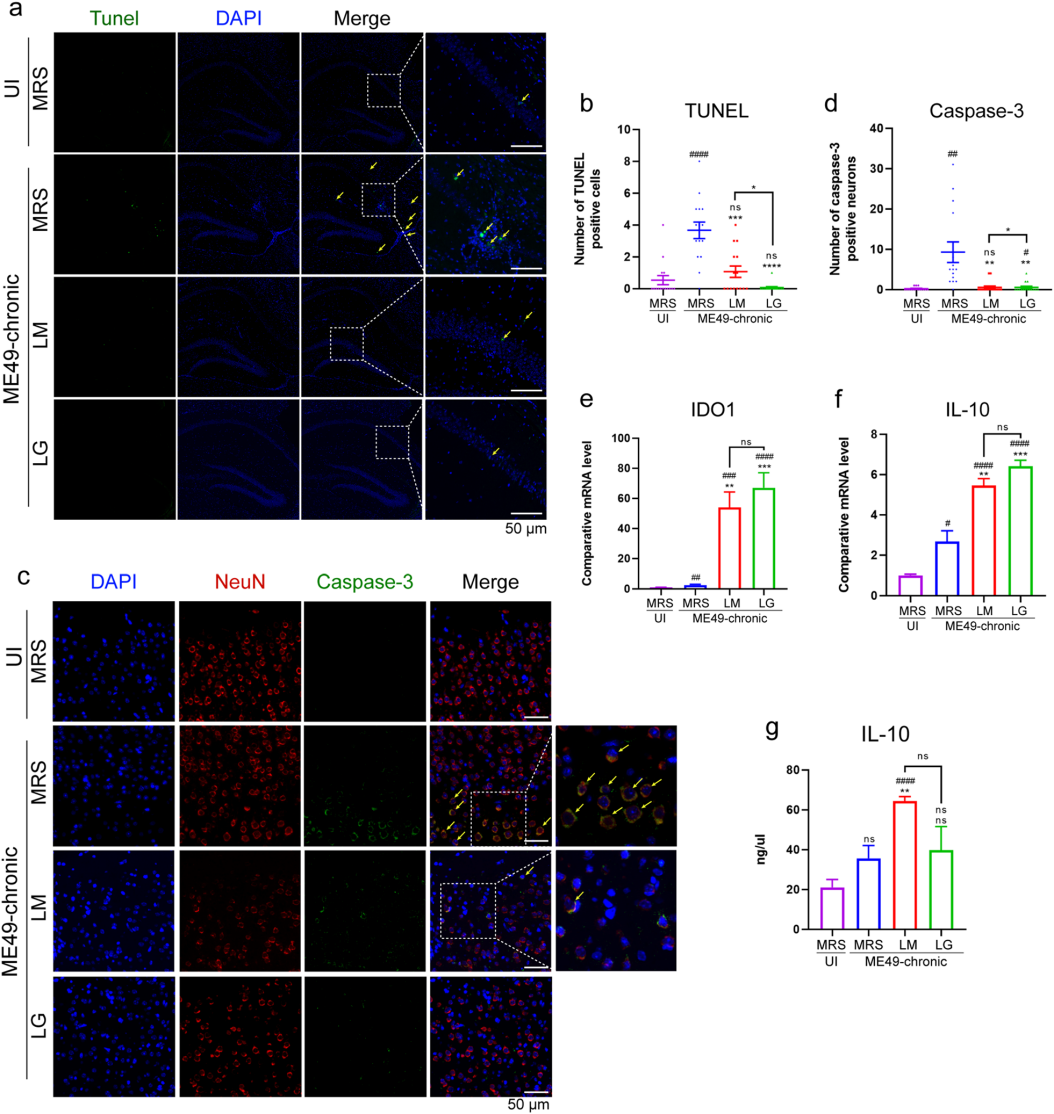
Detection of the therapeutic effect of *Lactobacillus* transplantation on neuron apoptosis during chronic infection. **a, b** Representative immunofluorescence images (**a**) and quantification (**b**) of TUNEL-FITC staining in chronically infected brain sections (*n* = 6). Scale bar = 100 μm. **c**, **d** Representative anti-NeuN/anti-caspase-3 immunostaining (**c**) and quantification of fluorescence intensity (**d**) of caspase-3 expression (*n* = 6). The positive staining is indicated by yellow arrows. The fluorescence intensity and positive counting were measured in randomly selected high-power fields. Scale bar: 50 µm. **e**, **f** Transcription level of IDO1 (**e**) and IL-10 (**f**) in the brain at 30 dpi was detected using RT-qPCR (*n* = 6). **g** ELISA was used to detect the concentration of serum IL-10 at 30 dpi (*n* = 6). Statistical significance is determined using one-way ANOVA with Tukey’s multiple comparison test. The hashtags (#) indicate significant differences in comparison with the UI-MRS group, and the asterisks (*) indicate significant differences in comparison with the ME49-MRS group: #/*P < 0.05, ##/**P < 0.01, ###/***P < 0.001, ####/****P < 0.0001; ns, not significant. ANOVA, Analysis of variance; FITC, fluorescein isothiocyanate; IDO, indoleamine 2,3-dioxygenase; IL, interleukin; ME49, cysts of the ME49 strain of* T. gondii*; MRS, DeMan, Rogosa and Sharpe culture medium; RT-PCR, reverse transcription PCR; TUNEL, terminal deoxynucleotidyl transferase dUTP nick end labeling; UI, uninfected; *vim−/−*, vimentin gene knockout mice; WT, wild-type mice

To investigate whether *Lactobacillus* transplantation ultimately alleviated neuronal apoptosis, TUNEL staining was employed to detect cell apoptosis in CNS in the WT mice at 30 dpi (Fig. 7a and b). The finding revealed that the overall number of TUNEL-positive cells at 30 dpi was lower compared to those at 90 dpi (Fig. 2a, b, 7a and b), with the ME49-MRS group exhibiting the highest number of TUNEL-positive cells (Fig. 7a, b). The results of immunofluorescence co-localization labeling for caspase-3 and NeuN indicated that, compared to un-infected and *Lactobacillus*-transplanted groups, the ME49-MRS group demonstrated the highest number of apoptotic cells (Fig. 7c, d).

IDO (indoleamine 2,3-dioxygenase) is a tryptophan metabolizing enzyme mainly secreted by M2-macrophages, which inhibits the proliferation of *T. gondii* by catalyzing the degradation of tryptophan necessary for its growth. By detecting the transcription levels of the gene encoding IDO (*ido1*) in the brain, we found that there was still a significant upregulation trend of IDO1 in the *Lactobacillus* transplantation group during the non-acute infection period (Fig. 7e). In addition, the transcription level of IL-10 in the *Lactobacillus* treatment group was also elevated (Fig. 7f). Compared to the other three groups, the ME49-LM group exhibited the highest levels of serum IL-10 (Fig. 7g).

### *Lactobacillus murinus* and *L. gasseri* transplantation facilitated CD8^+^T lymphocyte and M2-macrophage activation during *T. gondii* infection

**Fig. 8:**
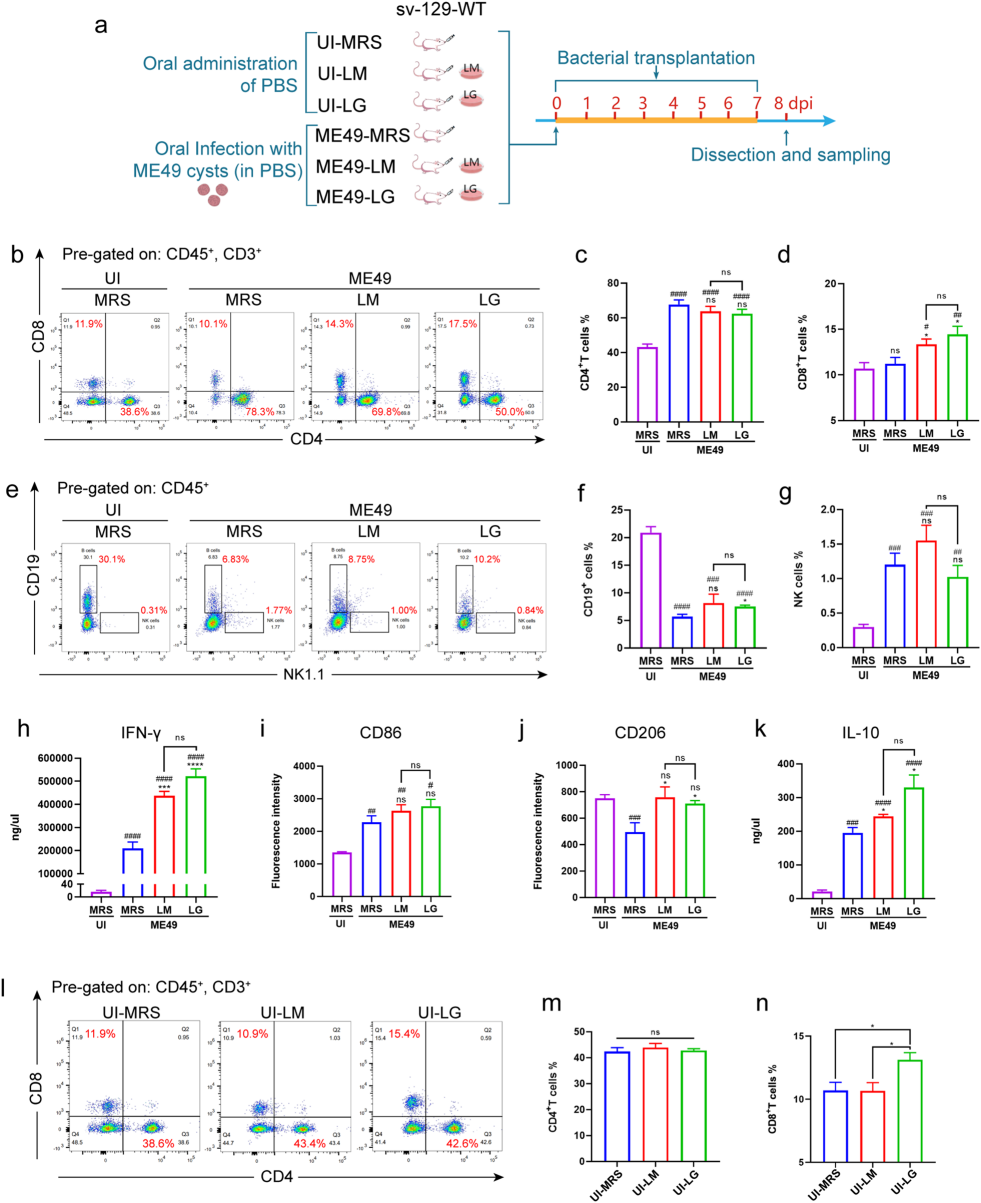
Detection of the regulation of two *Lactobacillus* strains on innate immune cells following transplantation. **a** Schematic of the experimental design. **b**–**g** Flow cytometry detection of the proportion of immunocytes in peripheral blood at 8 dpi, including CD4^+^T lymphocytes (**b**,** c**), CD8^+^T lymphocytes (**b**, **d**), B lymphocytes (**e**, **f**) and NK cells (**e**, **g**). **h** ELISA was used to detect the concentration of serum IFN-γ at 8 dpi. **i**, **j** Flow cytometry was used to detect the differentiation of macrophages in peritoneal fluid. The fluorescence intensity of CD86^+^ cells represent M1-macrophages (**i**), and CD206^+^ cells represent M2-macrophages (**j**). **k** ELISA was used to detect the concentration of serum IL-10 at 8 dpi. l-n Flow cytometry detection of the proportion of CD4^+^ (l, m) and CD8^+^T lymphocytes (l, n) after *Lactobacillus* transplantation in uninfected conditions. Statistical significance was determined using one-way ANOVA. The hashtags (#) indicate significant differences in comparison with the UI-MRS group, and the asterisks (*) indicate significant differences in comparison with the ME49-MRS group: #/*P < 0.05, ##/**P < 0.01, ###/***P < 0.001, ####/****P < 0.0001; ns, not significant. ANOVA, Analysis of variance; ELISA, enzyme-linked immunosorbent assay; dpi, days post-infection; IFN, interferon; IL, interleukin; ME49, cysts of the ME49 strain of* T. gondii*; MRS, DeMan, Rogosa and Sharpe culture medium; UI, uninfected; *vim−/−*, vimentin gene knockout mice; WT, wild-type mice

We speculated that the significant inhibitory effect of the two *Lactobacillus* strains on *T. gondii* may be attributed to their regulation of host immunity. Therefore, we detected the proportion of immunocytes associated with *T. gondii* infection in cases of infection or *Lactobacillus* transplantation (Fig. 8a). The results showed that *T. gondii* infection significantly promoted CD4^+^T and NK cell proliferation (Fig. 8b, c, e, g), while correspondingly, the proportion of B cells decreased (Fig. 8e, f). Specifically, *L. murinus* and *L. gasseri* transplantation enhanced the proliferation of CD8^+^T cells (Fig. 8b, d), while showing no significant effect on CD4^+^T cells and NK cells (Fig. 8c, g). Importantly, the concentration of IFN-γ, which is mainly secreted by CD8^+^T cells and has the strongest inhibitory effect on *T. gondii*, tended to double in the serum after *Lactobacillus* transplantation (Fig. 8h).

We further investigated the effects of *L. murinus* and *L. gasseri* transplantation on macrophage differentiation. The flow cytometry results revealed that *T. gondii* infection upregulated M1-macrophages (Fig. 8i; Additional file 5: Figure S5a) and that *L. murinus* and *L. gasseri* transplantation increased the amount of M2-macrophage (Fig. 8j; Additional file 5: S5a). The levels of IL-10 were consistent with the observed changes in M2-macrophage populations in *Lactobacillus* transplantation, showing significant upregulation in *T. gondii* infection and *Lactobacillus* transplantation (Fig. 8k). The levels of IL-10 consistent with the trend of brain transcription levels and serum levels during chronic infection.

Interestingly, without *T. gondii* infection, the administration of *L. gasseri* alone directly promoted the proliferation of CD8^+^ T cells (Fig. 8l–n), but *L. murinus* alone did not have this effect. In addition, the transplantation of *L. murinus* and *L. gasseri* alone did not affect the proportion of other immune cells (Fig. 8m; Additional file 5: Figure S5b–g), indicating that the activation of CD8^+^ T cells is directly regulated by *L. gasseri* or its metabolites.

In addition, without infection, transplantation of *L. murinus* could directly promote M2-polarization of macrophages (Addition file 5: Figure S5e, f). These findings are in line with previous reports on the promotion of M2-macrophage differentiation and IL-10 secretion [56]. This may be attributed to *T. gondii* infection promoting the activation of the TLR2 signaling pathway in macrophages [59].

These results also suggest that these two types of lactobacilli have different immune regulatory abilities or may trigger different signaling pathways.

### *Toxoplasma gondii *infection resulted in a decrease in metabolites derived from lactobacilli in the serum

**Fig. 9:**
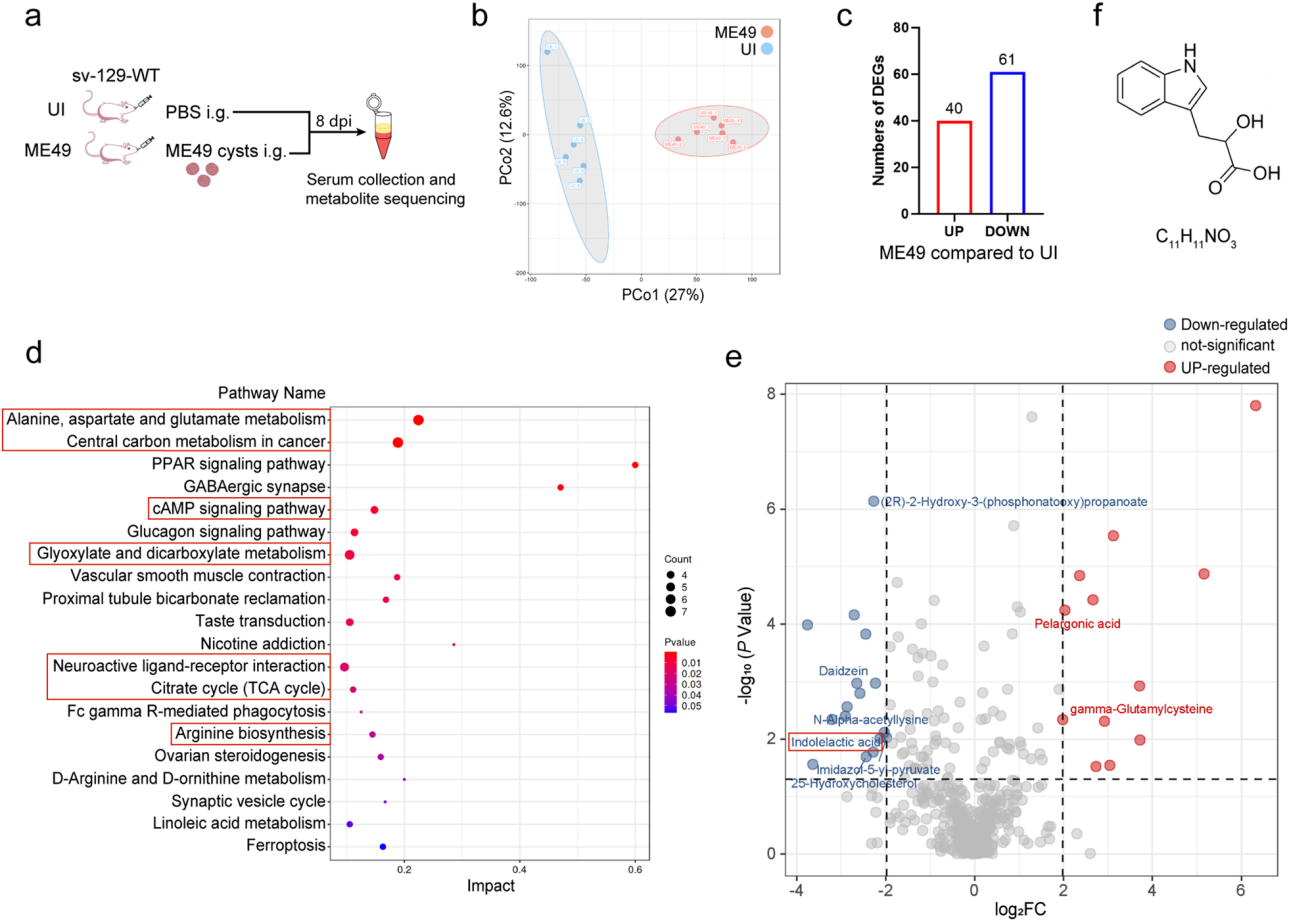
Detection of the effect of *T. gondii* infection on serum small molecule metabolites by untargeted metabolomics sequencing. **a** Schematic of the experimental design. **b** PCoA analyses between two groups of mice at 8 dpi. **c** Number of differential metabolites detected with fold change ≥ 10 (|Log_2_FC|≥ 2) and *P* value ≤ 0.05. **d** KEGG signaling pathway analysis for the differential metabolites. **e** Volcano plot of differential metabolites with representative *Lactobacillus* metabolites are indicated. **f** The structural and molecular formulas of ILA. DEG, Differentially expressed genes; dpi, days post-infection; ILA, indole-3-lactic acid; KEGG, Kyoto Encyclopedia of Genes and Genomes; ME49, cysts of the ME49 strain of* T. gondii*; PBS, phosphate-buffered saline; PCoA principal component analysis; UI, uninfected

To elucidate the impact of *T. gondii* infection on circulating metabolites, serum samples were collected at 8 dpi for comprehensive targeted metabolomic sequencing (Fig. 9a). The results of principal coordinates analysis (PCoA) revealed that *T. gondii* infection led to significant alterations in the composition of metabolites (Fig. 9b). Specifically, 40 substances were upregulated, while 61 substances were downregulated (Fig. 9c). The differentially regulated metabolites were found to be enriched in signal pathways primarily associated with energy and amino acid metabolism, including GABAergic signaling pathways closely linked to neurological diseases, which were significantly upregulated following infection (Fig. 9d). Notably, a variety of metabolites or metabolic intermediates derived from *Lactobacillus *were detected among these altered metabolites, as indicated in the volcano plot, with most of these substances experiencing significant downregulation after infection (Fig. 9e).

Among these, there is an aromatic compound in the tryptophan metabolism pathway called indole-3-lactic acid (ILA; Fig. 9e, f). Previous studies have shown that ILA can activate the aromatic hydrocarbon receptor (AhR) signaling pathway, thereby regulating immune cells such as CD4^+^ and CD8^+^ T cells [60]. Therefore, we hypothesized that these two strains of lactobacilli might activate the AhR signaling pathway by secreting ILA.

### *Lactobacillus gasseri* show a stronger ability to metabolize ILA, which can promote CD8^+^ T cell proliferation by activating the AhR pathway

**Fig. 10:**
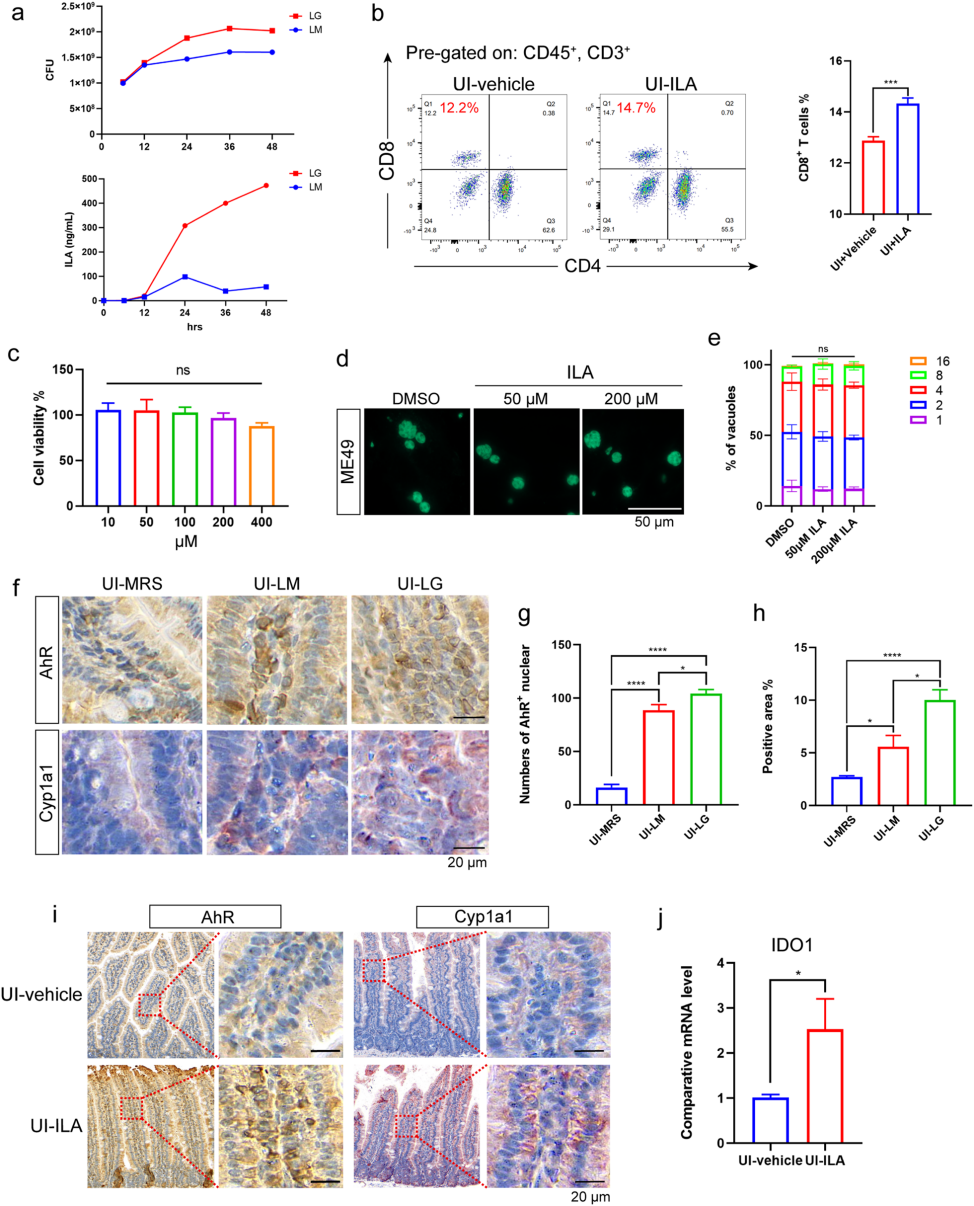
Detection of the effects of the two *Lactobacillus* strains and ILA on the activation of AhR signaling pathway in IECs. **a** Detection of ILA secretion ability by *L. murinus* and *L. gasseri*: growth rate of *L. murinus* and *L. gasseri* with the same initial CFU (above) and concentration of ILA in the culture medium (below). **b** Flow cytometry detection of the proportion of CD4^+^ and CD8^+^T lymphocytes in peripheral blood after 8 days of ILA administration. **c** CCK8 experiment to detect the effect of different concentrations of ILA on the viability of HFF cells. **d**-**e** Detection of the effect of ILA on the proliferation of *T. gondii* ME49 tachyzoites under oil microscopy (**d**); intracellular replication rates of ME49 with or without lLA treatment, as determined by the distribution of parasitophorous vacuoles (PVs) containing l, 2, 4, 8 or 16 parasites after 24 h of intracellular growth (**e**). Data are presented as the mean ± SEM of *n* = 4 independent experiments. **f-i** Detection of the effects of *L. murinus* and *L. gasseri* transplantation therapy (**f**–**h**) and ILA therapy (**i**) on AhR nuclear metastasis (umber) and cyp1a1 expression level (red) in the jejunum. **j** Transcription level of *ido1* in jejunum. Quantitative analysis was performed by calculating the number of AhR^+^ cells co-located with the nucleus, or by counting the positive area of cyp1a1 in randomly selected high-power fields. Statistical significance was determined using unpaired *t*-test (**b**, **j**), or one-way ANOVA (**c**, **g**, **h**), or two-way ANOVA with Tukey’s multiple comparisons post-tests (**e**). Asterisks (*) indicate significant differences at **P < 0.01, ***P < 0.001, ****P < 0.0001; ns, not significant. AhR, Aromatic hydrocarbon receptor; ANOVA, analysis of variance; CCK8, Cell Counting Kit 8; CFU, colony-forming unit; Cyp, cytochrome P450; HFF, human foreskin fibroblasts; ICEs, intestinal epithelial cells; IDO, indoleamine 2,3-dioxygenase; ILA, indole-3-lactic acid; LG, *Lactobacillus gasseri*; LM, *Lactobacillus murinus*; MRS, DeMan, Rogosa and Sharpe culture medium; SEM, standard error of the mean; UI, uninfected

We firstly evaluated the ILA secretion ability of *L. murinus* and *L. gasseri* (Fig. 10a). Surprisingly and as expected, the results showed that *L. gasseri* exhibited a stronger ability to secrete ILA than *L. murinus* (Fig. 10a). Additionally, after 8 days of continuous gastric administration of ILA, we observed an increase in the number of CD8^+^ T cells in mice (Fig. 10b). The cell model experiments indicated that ILA itself does not directly inhibit the proliferation of *T. gondii* (Fig. 10c–e).

Furthermore, our findings demonstrated that both *L. gasseri* transplantation and ILA administration can promote the nuclear translocation of AhR in IECs (Fig. 10f, g, i), indicating activation of the AhR signaling pathway. In addition, the expression level of downstream cytochrome P450 family protein, CYP1a1, and the transcription level of *ido1* were significantly increased after ILA treatment (Fig. 10f, h, i, j). After treating with the AhR inhibitor CH223191, the activating effect of *L. gasseri* on CD8^+^ T cells disappeared (Additional file 5: Figure S5h, i).

## Discussion

**Fig. 11:**
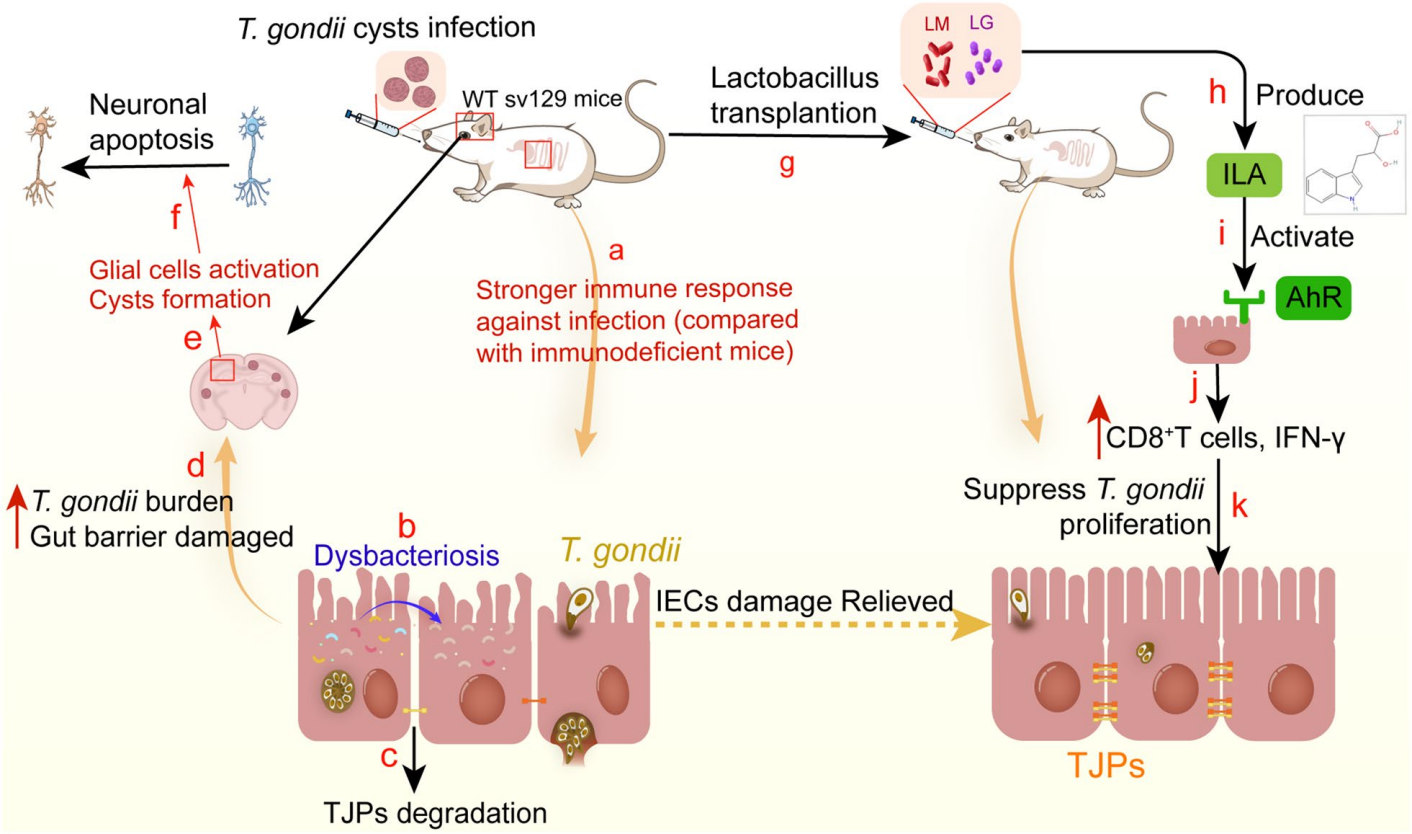
Mechanisms underlying the alleviation of intestine and brain damage during *T. gondii* infection by the transplantation of two *Lactobacillus* strains. **a**, **b** After oral infection with *T. gondii* cysts, WT mice showed significant disruption in their gut microbiota due to a more severe immune response, leading to a decrease in the abundance of various lactobacilli. **c** ME49 tachyzoites invade and multiply within the IECs, resulting in small intestinal mucosal damage characterized by inflammation infiltration, villi shortening, increased crypt depth and degradation of TJPs between IECs. **d**–**f** Consequently, *T. gondii* spreads to extra-intestinal organs due to intestinal barrier damage (**d**), resulting in an increased cyst formation rate in the brain and subsequent activation of microglia and astrocytes (**e**), which results in neuron apoptosis manifesting as neurodegenerative symptoms in mice (**f**). **g**-**h** Supplementation of WT mice with *Lactobacillus murinus* and *L. gasseri* during infection significantly ameliorates fatal enteritis and chronic brain injury (**g**); *Lactobacillus murinus* and *L. gasseri* are capable of secreting ILA, with *L. gasseri* exhibiting a stronger ILA secretion ability (**h**). **i** ILA activates the AhR signaling pathway in intestinal lamina propria cells. **j** This activation promotes the activation of CD8^+^T cells and secretion of IFN-γ. **k** Consequently, *T. gondii* proliferation is significantly inhibited. AhR, Aromatic hydrocarbon receptor; IECs, intestinal epithelial cells; ILA, indole-3-lactic acid; INF, interferon; LG, *Lactobacillus gasseri*; LM, *Lactobacillus murinus*; TJPs, tight junction proteins; WT, wild type

Previous studies have reported that mice infected with *T. gondii* exhibit cognitive impairments, such as decreased learning ability and memory, as well as a tendency towards depression, characterized by reduced anxiety and social interaction [58, 61–64]. These behavioral changes are accompanied by significant alterations in the composition of the intestinal flora. Using an animal model of immune-compromised mice, *vim−/−* mice, we further demonstrated that these psychiatric and degenerative lesions, as well as dysbiosis of the flora, are closely linked to the host immune response in clearing the infection. The flora transplantation experiment provided additional evidence that the severity of CNS lesions is closely associated with the balance of intestinal lactobacillus. The reduction of in the level of various metabolites produced by *Lactobacillus* strains, particularly those related to the tricarboxylic acid cycle or amino acid metabolism in the serum (Additional file 6: Table S1.), plays a crucial role in regulating the “gut-brain axis” (Fig. 11).

Our investigation further revealed that the population of apoptotic neurons in the brains of mice experiencing chronic *T. gondii* infection for a duration of 3 months was notably higher compared to that observed in the 1-month infection group. This observation suggests a progressive colonization of *T. gondii* in the brain over time, potentially attributed to the sustained activation of microglia and astrocytes. These findings underscore the importance of early parasite elimination, in particular when the current absence of specific drugs for targeting *T. gondii* cysts is taken into account.

Analysis of the brain transcriptome revealed that the expression of DEGs primarily involved antigen presentation and the immune responses of T lymphocytes, indicating the immunocompromised status in *vim−/−* mice but a less severe gut inflammation response to *T. gondii* infection. Indeed, the immune pressure exerted by WT mice during the clearance of *T. gondii* may inadvertently exacerbate disturbances in the gut microbiota. The divergence in T-lymphocyte activation may be attributed to the absence of vimentin, a protein that plays a crucial role in lymphocyte homing, maturation and differentiation [39]. Alternatively, this variation could also be influenced by the differing burden of *T. gondii* cysts reflected through gut-brain axis (Fig. 11). *Toxoplasma gondii*-secreted proteins significantly altered the quantity and composition of gut microbiota [65]. Additionally, *T. gondii* triggered cytokine production and T-cell activation through the TLR pathway, leading to the depletion of Paneth cells in IECs and a subsequent decrease in the secretion of anti-inflammatory substances, thereby exacerbating enteritis and causing damage to the intestinal lamina propria [66]. Interestingly, studies have demonstrated that germ-free mice do not exhibit intestinal inflammation after *T. gondii* infection [67]. Additionally, infection with *T. gondii* in sterile animals has been shown to increase their survival rate [66], suggesting a role for gut microbiota in the pathogenesis of toxoplasmosis.

The gut microbiota plays a crucial role in the maturation of the intestinal epithelial barrier and the development of intestinal immunity [68], contributing to the regulation of the mucus layer, the development of immune cells in the intestinal lamina propria and the activation and differentiation of various lymphocyte groups [69]. Notably, research has demonstrated that *L. gasseri* can activate the NF-kappa B pathway and the oxidative phosphorylation pathway to promote immune regulation and energy metabolism in porcine IECs [70]. Additionally, *L. gasseri* produces antimicrobial peptides and metabolites, such as lactic acid and bacteriocins, which exhibit antibacterial activity and may downregulate the expression of virulence factors in intestinal pathogens [52, 71, 72].

Intraepithelial lymphocytes (IELs) are activated by various pattern recognition receptors, including lipopolysaccharides, flagellar proteins and bacterial peptidoglycans [73]. They play a role in regulating immune responses against intestinal pathogens and symbiotic microbiota [68]. These findings suggest that *L. murinus* and *L. gasseri* may share conserved antigenic epitopes that activate CD8^+^ T cells, providing valuable insights for further exploration of the immunomodulatory effects of these *Lactobacillus* strains.

However, the relationship between specific pathogens and gut microbiota disorders can vary depending on the pathogen and the host’s background. Although our research elucidated the immunomodulatory effects of lactobacilli during *T. gondii* colonization, it is noteworthy that the effects of the two bacterial species are not entirely identical. Of the two, *L. murinus* exhibits the more prolonged activation effect on M2-macrophages, independent of infection factors, while *L. gasseri* appears to possess regulatory effects on CD8^+^ T cells and B cells. These differences may be caused by variations in the secretion levels of ILA by these two *Lactobacillus* strains or by metabolic products.

In the metabolomic analysis, several metabolites produced by *Lactobacillus*, such as *N*-alpha-acetyl-lysine and imidazole-5-yl-pyruvate, were significantly downregulated after infection, as shown in Additional file 6: Table 1. These metabolites play a crucial role in bridging the gap between the gut microbiota and the brain, regulating the inflammatory response, antioxidant level and immune cell activation [74, 75]. For example, ILA, produced by *L. plantarum*, has been shown to reduce colorectal tumorigenesis through epigenetic regulation of CD8^+^ T-cell immunity [74]. Many of these l-tryptophan metabolites act as agonists of AhR, directly activating the AhR signaling pathway in IELs, thereby regulating IEC proliferation and controlling the inflammatory response [76, 77]. Pyruvate and l-lactate, metabolites of *Lactobacillus*, have also been shown to enhance cellular resistance to oxidative stress and reduce cell death by activating the unfolded protein response and nuclear factor erythroid 2-related factor 2 [78].

The metabolomic analysis also revealed downregulation of SCFAs, including *N*-acetylserotonin, arachidonic acid, kynurenic acid, acetylcholine and gamma-aminobutyric acid, as shown in Additional file 6: Table 1. These SCFAs can directly influence the secretion of intestinal hormones, which, in turn, can affect the production of neurotransmitters. On the other hand, SCFAs can provide energy and promote the growth of bacteria such as *Escherichia coli*, *Lactobacillus* and *Bifidobacterium*. This suggests that the metabolites produced by *L. murinus* and *L. gasseri*, rather than the bacteria themselves, are the primary factors influencing immune regulation.

Transplantation of the resident gut microbiota has emerged as an innovative treatment strategy for various intestinal diseases [66, 79]. Based on the present study, we conclude that correcting the decrease of *L. murinus* and *L. gasseri* caused by infection in WT mice is a promising therapeutic approach. ILA is also a small molecule drug with great potential for treating *T. gondii* infection. Transplantation of these *Lactobacillus* strains or ILA effectively alleviated the lethal enteritis and brain damage caused by *T. gondii* infection.

## Conclusions

Our study demonstrates that the host immune response can exacerbate infection-induced damage during *T. gondii* infection. Notably, infected mice exhibited more severe small intestinal tissue damage, prolonged tissue injury, heightened cerebral inflammation and increased neuronal apoptosis compared to immunocompromised mouse models. Consequently, the chronic *T. gondii* infected mice displayed impaired cognitive abilities and pronounced depressive tendencies. These outcomes are attributed to dysbiosis of the gut microbiota during the infection process. Importantly, supplementation with *L. murinus* and *L. gasseri* increased the proportion of CD8^+^ T cells in peripheral blood circulation and the concentration of serum IFN-γ, leading to a significant reduction in the *T. gondii* burden across multiple organs. These beneficial effects are mediated through the secretion of ILA by these *Lactobacillus* strains and activation of the AhR signaling pathway in intestinal epithelial cells.

### Supplementary Information

**Additional file 1: Figure S1.** Detection of CNS pathology in WT and *vim−/−* mice in chronic infection by behavioral testing and transcriptome sequencing.

**Additional file 2: Figure S2.** Evaluation of the therapeutic effect of *L. murinus* and *L. gasseri* transplantation on the jejunum, ileum, and cecum after *T. gondii* infection.

**Additional file 3: Figure S3.** Detection of the effect of *L. murinus* and *L. gasseri* treatment on the repair of *T. gondii* infected small intestine.

**Additional file 4: Figure S4.** Detection of the alleviating effect of *L. murinus* and *L. gasseri* transplantation on the activation of CNS glial cells during chronic infection.

**Additional file 5: Figure S5.** Effects of the transplantation of two *Lactobacillus* strains on modulation of host immune cells with or without *T. gondii* infection.

**Additional file 6: Table S1.** Differential serum metabolite molecules detected by whole target metabolite sequencing between UI and ME49 infected mice.

## Data Availability

The datasets supporting the findings of this article are included within the paper and its supplementary materials. The RNA-seq raw data described in the present study has been submitted to the NCBI Sequence Read Archive database with the accession number PRJNA1071334.

## Abbreviations

AIDS: Acquired immunodeficiency syndrome
AhR: Aromatic hydrocarbon receptor
BBB: Blood–brain barrier
CNS: Central nervous system
DCs: Dendritic cells
HIV: Human immunodeficiency virus
HE: Hematoxylin and eosin
IBD: Inflammatory bowel disease
IECs: Intestinal epithelial cells
IELs: Intraepithelial lymphocytes
IFN: Interferon
IL: Interleukin
ILA: Indole-3-lactic acid
LM: *L* *.* *murinus*
LG: *L. gasseri*
MHC: Major histocompatibility
MRS medium: DeMan, Rogosa and Sharpe culture medium
NO: Nitric oxide
PERK: Protein kinase R-like endoplasmic reticulum kinase
PBS: Phosphate-buffered saline
RI: Recognition index
SCFAs: Short-chain fatty acids
SPF: Specific pathogen-free
TJPs: Tight junction proteins
TLR: Toll-like receptor
TNF: Tumor necrosis factor
TUNEL: Terminal deoxynucleotidyl transferase dUTP nick end labeling
WT: Wild type
