# Supplementary figures and images for: Intestinal microbiota imbalance resulted by anti-Toxoplasma gondii immune responses aggravate gut and brain injury
Source: Parasit Vectors. 2024 Jul 2;17:284. doi: 10.1186/s13071-024-06349-8 (PMC11221008; doi:10.1186/s13071-024-06349-8)

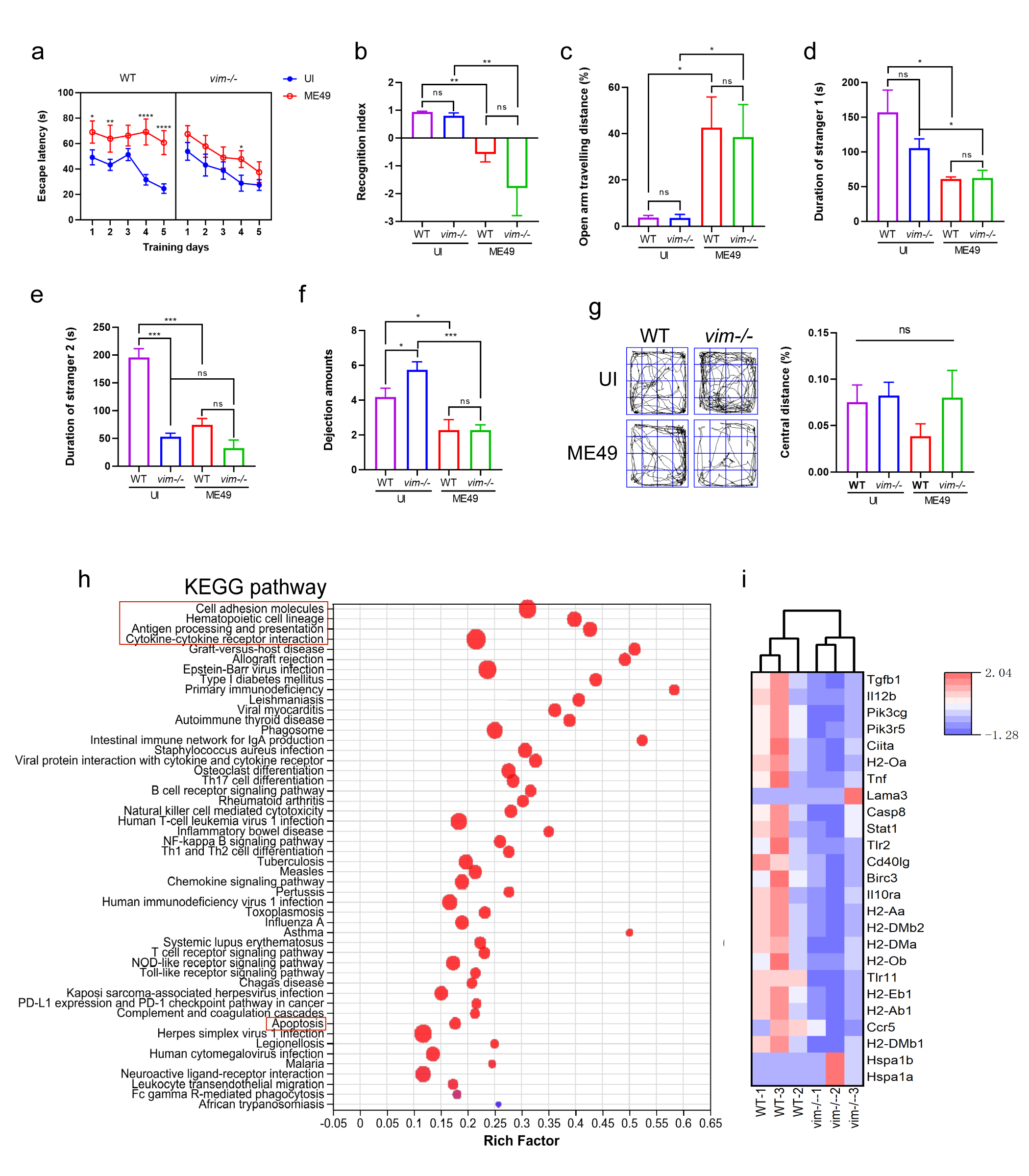

Supplement: Supplementary file 1 — Additional file 1: Figure S1. Detection of CNS pathology in WT and vim−/− mice in chronic infection by behavioral testing and transcriptome sequencing. [file 13071_2024_6349_MOESM1_ESM.tif]

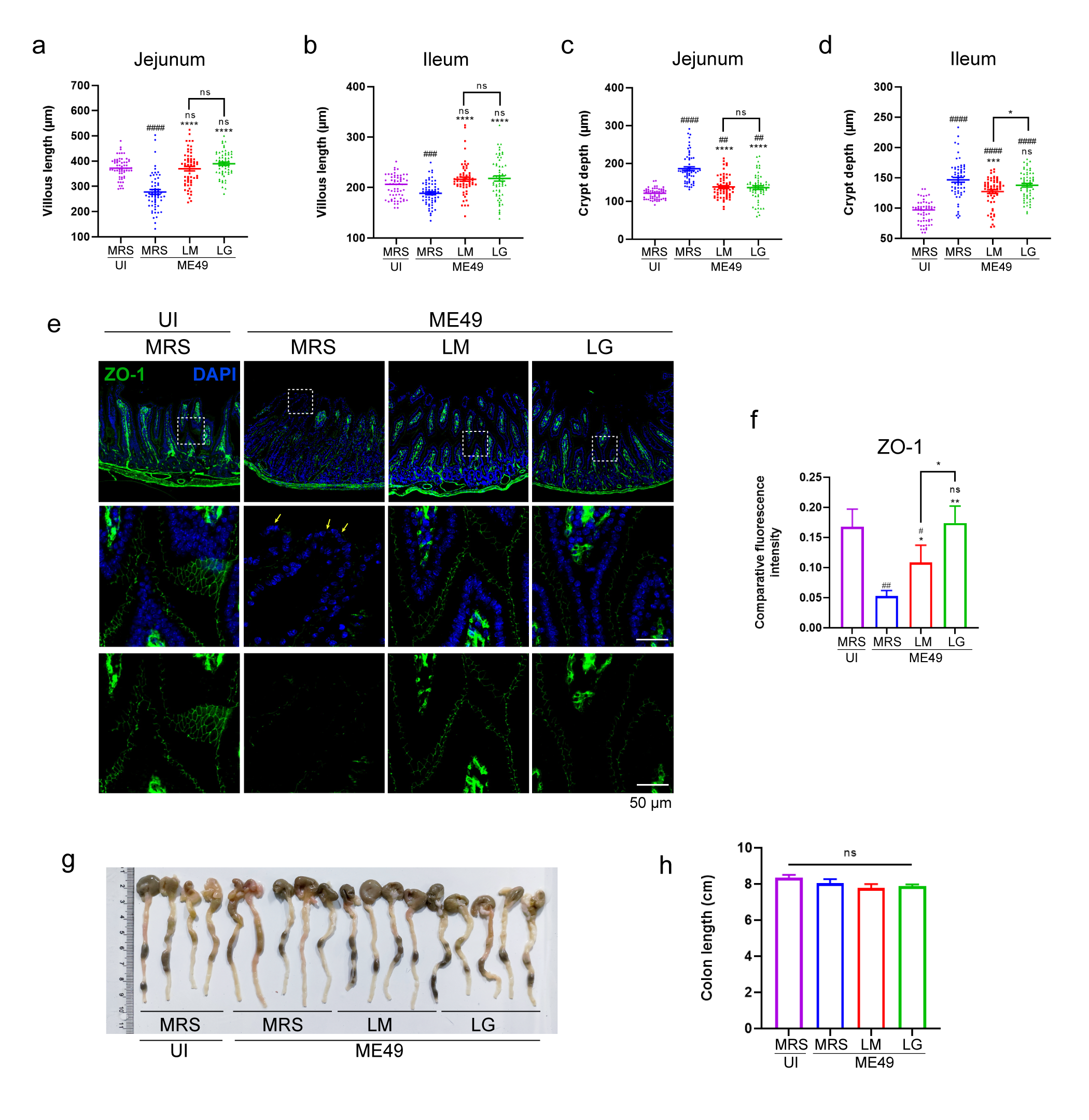

Supplement: Supplementary file 2 — Additional file 2: Figure S2. Evaluation of the therapeutic effect of L. murinus and L. gasseri transplantation on the jejunum, ileum, and cecum after T. gondii infection. [file 13071_2024_6349_MOESM2_ESM.tif]

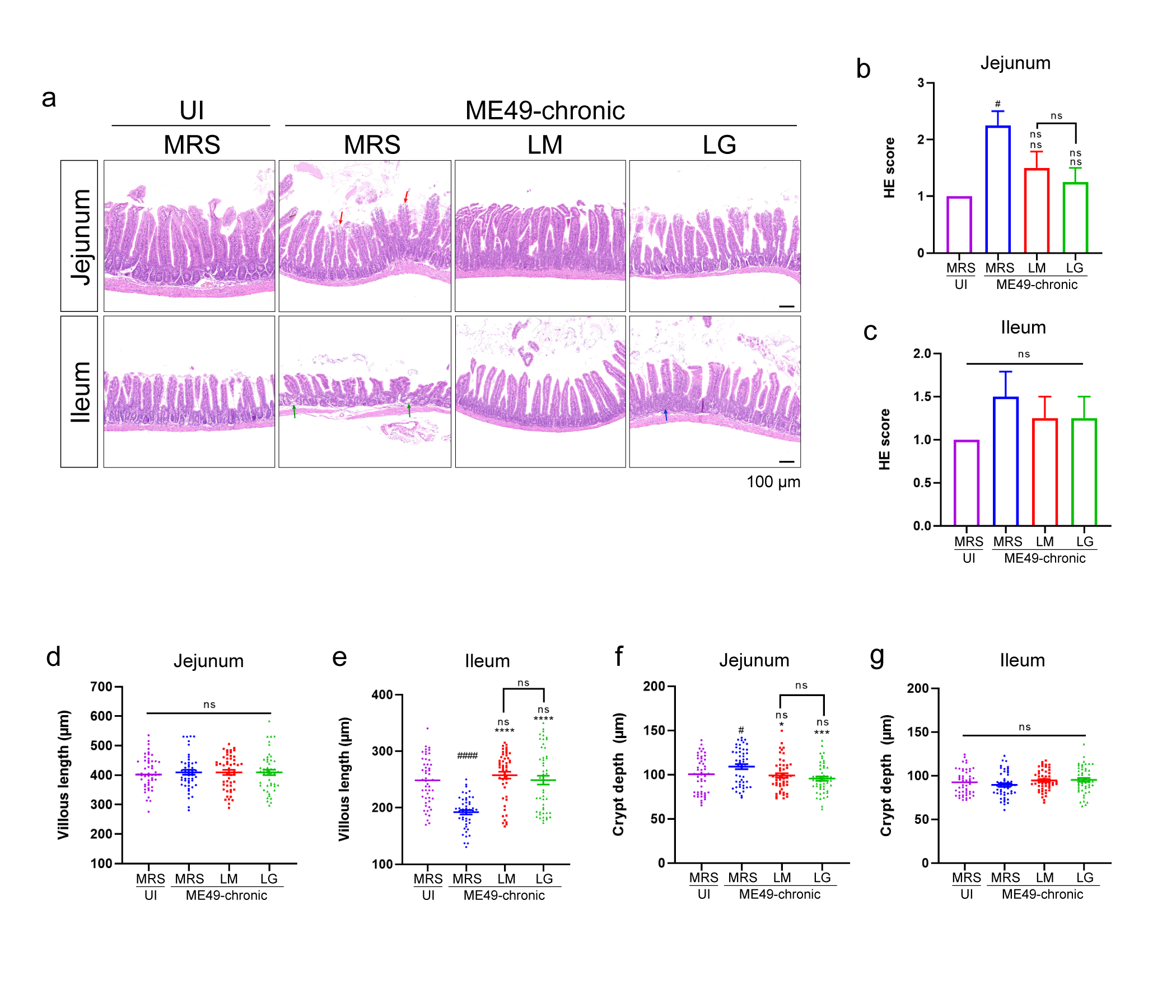

Supplement: Supplementary file 3 — Additional file 3: Figure S3. Detection of the effect of L. murinus and L. gasseri treatment on the repair of T. gondii infected small intestine. [file 13071_2024_6349_MOESM3_ESM.tif]

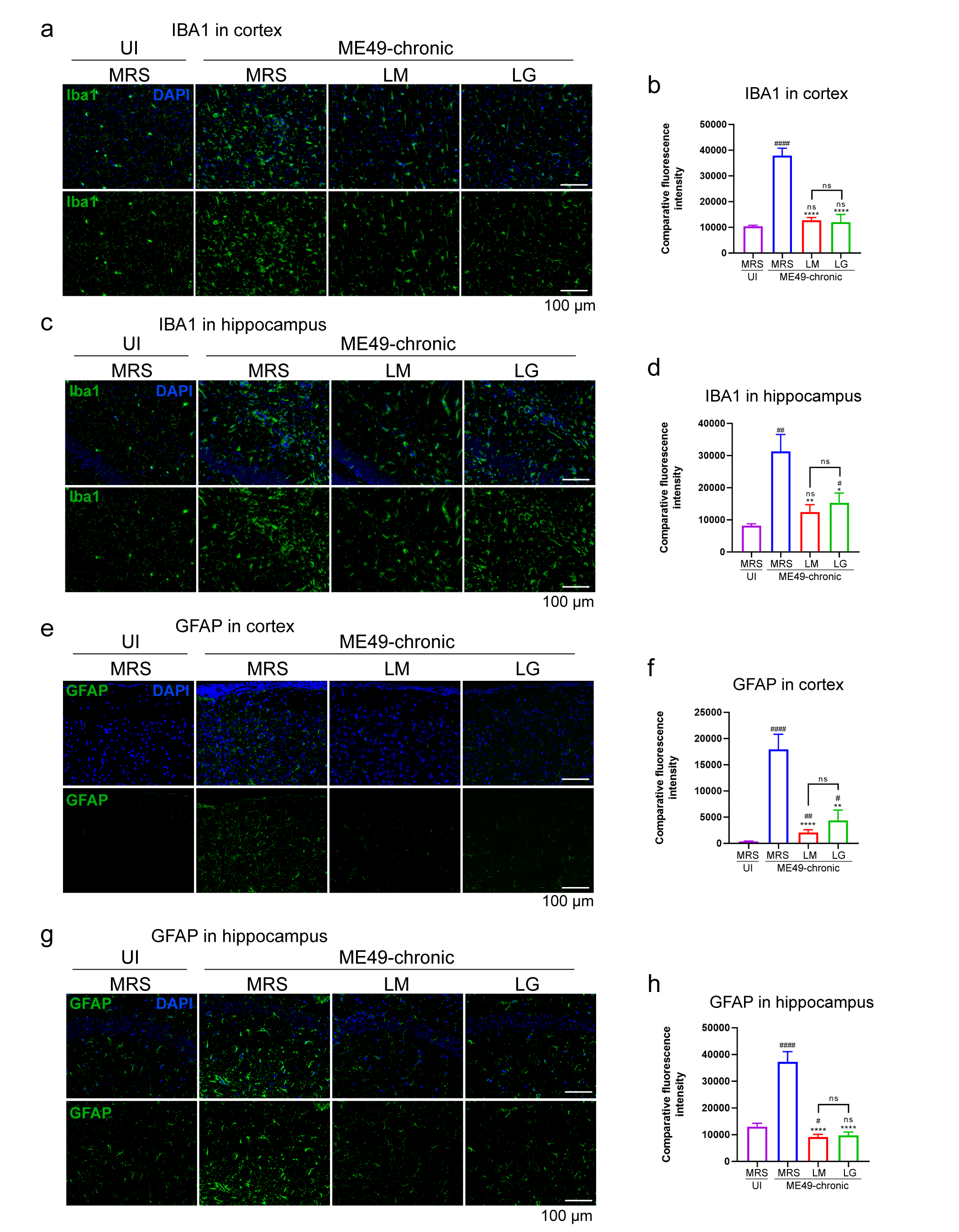

Supplement: Supplementary file 4 — Additional file 4: Figure S4. Detection of the alleviating effect of L. murinus and L. gasseri transplantation on the activation of CNS glial cells during chronic infection. [file 13071_2024_6349_MOESM4_ESM.tif]

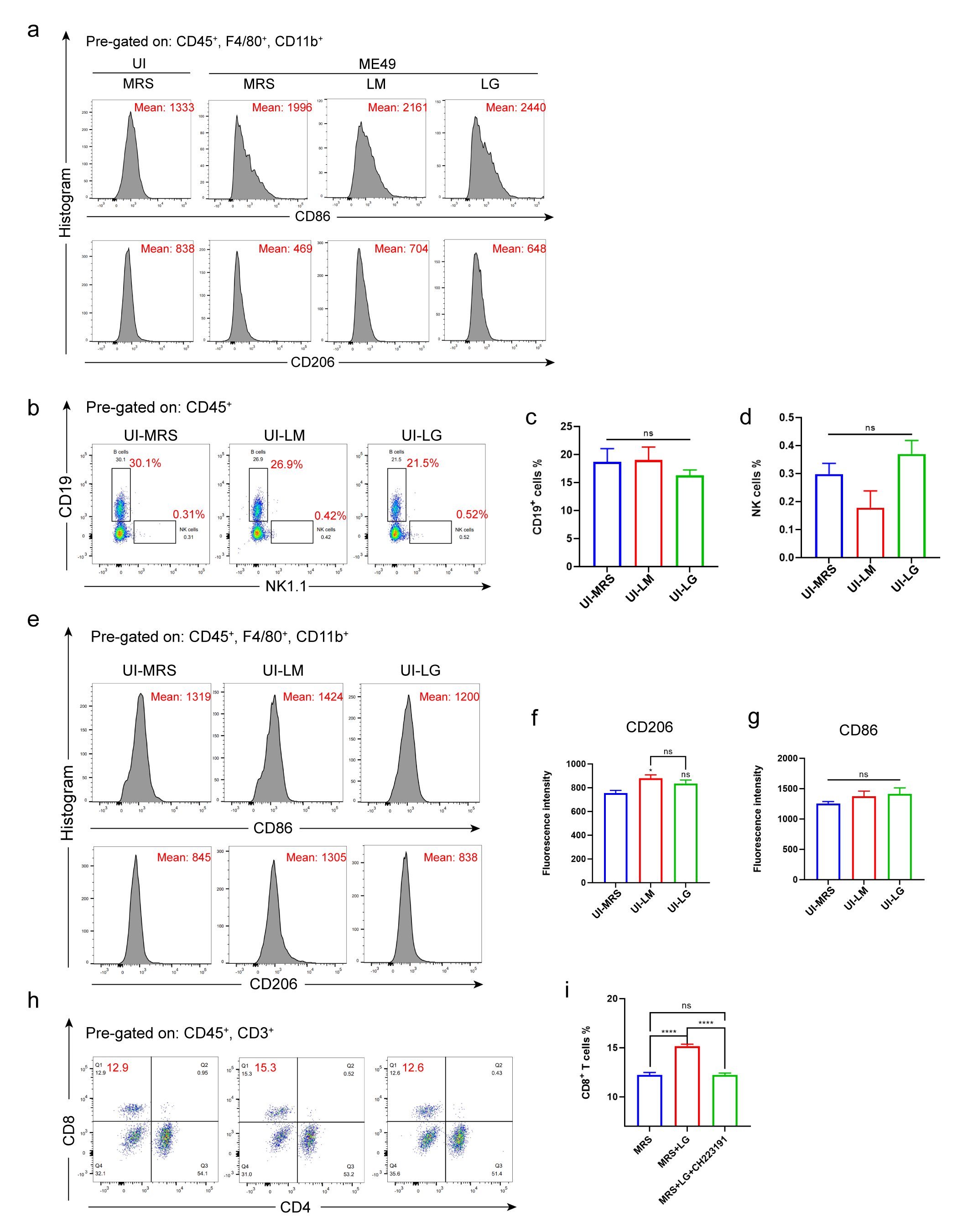

Supplement: Supplementary file 5 — Additional file 5: Figure S5. Effects of the transplantation of two Lactobacillus strains on modulation of host immune cells with or without T. gondii infection. [file 13071_2024_6349_MOESM5_ESM.tif]
