# Supplementary material for: Intestinal microbiota imbalance resulted by anti-Toxoplasma gondii immune responses aggravate gut and brain injury
Source: Parasit Vectors. 2024 Jul 2;17:284. doi: 10.1186/s13071-024-06349-8 (PMC11221008; doi:10.1186/s13071-024-06349-8)
Supplement: Supplementary file 6 — Additional file 6: Table S1. Differential serum metabolite molecules detected by whole target metabolite sequencing between UI and ME49 infected mice. [file 13071_2024_6349_MOESM6_ESM.docx]

**Supplementary table**

**Table S1**

**Differential serum metabolite molecules detected by whole target metabolite sequencing between UI and ME49 infected mice.**

| **Name** | **Log_2_FC** | ***P* value** | **ID** |
| --- | --- | --- | --- |
| Pyridoxamine | 6.32 | 1.58E-08 | M169T99 |
| 12-Ethyl-8-propyl-3-vinylbacteriochlorophyllide d | 5.16 | 1.34E-05 | M584T418_1 |
| N-Acetyl-aspartyl-glutamic acid | 3.72 | 0.010362 | M303T40 |
| Palmitoyl-L-carnitine | 3.71 | 0.001187 | M400T496_2 |
| Dehydroepiandrosterone | 3.12 | 2.91E-06 | M288T425 |
| Hydroquinone | 3.04 | 0.028598 | M111T33 |
| gamma-Glutamylcysteine | 2.92 | 0.004885 | M249T51 |
| 8,12-Diethyl-3-vinylbacteriochlorophyllide d | 2.73 | 0.030022 | M570T499_1 |
| o-Toluate | 2.66 | 3.79E-05 | M135T290 |
| N-Acetylleucine | 2.36 | 1.43E-05 | M174T279_2 |
| Pelargonic acid | 2.03 | 5.74E-05 | M158T427 |
| Sorbitol | 1.98 | 0.004576 | M183T266 |
| 8-Amino-7-oxononanoate | 1.9 | 0.001363 | M170T272 |
| Oxidized glutathione | 1.52 | 0.022917 | M613T72_3 |
| Alpha-dimorphecolic acid | 1.47 | 0.018074 | M279T553 |
| L-Arginine | 1.42 | 0.006382 | M175T59 |
| Oleic acid | 1.29 | 2.48E-08 | M283T37 |
| 15-Deoxy-d-12,14-PGJ2 | 1.27 | 0.022076 | M317T476 |
| Sebacic acid | 1.1 | 0.018108 | M185T320 |
| 3-Hydroxyanthranilic acid | 1.04 | 0.012939 | M154T51 |
| L-Homophenylalanine | 1.03 | 0.011245 | M180T232 |
| Stearolic acid | 1.03 | 6.14E-05 | M281T527 |
| Equol | 0.96 | 4.98E-05 | M243T321 |
| Chlorhexidine gluconate | 0.88 | 1.94E-06 | M196T46_2 |
| L-2-Amino-6-oxoheptanedioate | 0.85 | 0.000148 | M190T359 |
| Neocnidilide | 0.78 | 0.014941 | M177T330 |
| Antibiotic JI-20A | 0.78 | 0.012448 | M482T559 |
| Homocitrulline | 0.74 | 0.001363 | M190T73 |
| L-Olivosyl-oleandolide | 0.73 | 0.014347 | M517T478_1 |
| (9Z,11E,13E)-Octadecatrienoic acid | 0.58 | 0.011759 | M279T466 |
| 20-HETE | 0.59 | 0.003903 | M319T539 |
| Propionylcarnitine | 0.56 | 0.011194 | M218T82 |
| Creatinine | 0.42 | 0.003296 | M114T50 |
| 2-Hydroxybutyric acid | 0.42 | 0.016492 | M104T180 |
| 3-(3,4-Dihydroxyphenyl)pyruvate | 0.24 | 0.004419 | M197T54 |
| 16-Hydroxy hexadecanoic acid | 0.23 | 0.024762 | M255T507 |
| (R)-3-Hydroxybutyric acid | 0.21 | 0.000242 | M103T50_2 |
| Glyceric acid | 0.18 | 0.001559 | M105T46 |
| 9-cis-Retinoic acid | 0.16 | 0.03268 | M299T479 |
| 2-Pyrocatechuic acid | 0.15 | 0.004557 | M153T240 |
| Chavicol | -0.33 | 0.002278 | M135T472 |
| Lanosterin | -0.37 | 0.006311 | M425T516 |
| L-Malic acid | -0.46 | 0.01434 | M133T44 |
| 13S-hydroxyoctadecadienoic acid | -0.56 | 0.03207 | M295T537 |
| Dodecanedioic acid | -0.6 | 0.046665 | M213T386 |
| (2S)-2-{[1-(R)-Carboxyethyl]amino}pentanoate | -0.61 | 0.048617 | M188T70 |
| Guanosine | -0.65 | 0.011667 | M284T72 |
| Rutin | -0.69 | 0.004275 | M610T660 |
| Docosahexaenoic acid | -0.72 | 0.000508 | M329T550 |
| Sphingosine 1-phosphate | -0.82 | 0.003227 | M378T479_1 |
| Butyryl-L-carnitine | -0.86 | 0.010522 | M232T149 |
| Ciliatine | -0.88 | 0.008792 | M126T42 |
| gamma-Aminobutyric acid | -0.91 | 3.88E-05 | M104T74 |
| Capsidiol | -0.91 | 0.004803 | M219T372 |
| Methoprene | -0.9 | 0.012144 | M293T583 |
| 1,5-Naphthalenediamine | -0.94 | 0.001012 | M159T298 |
| L-Glutamine | -0.98 | 0.000356 | M147T50 |
| Niacinamide | -1.07 | 0.024595 | M123T54_2 |
| N-Acetyl-L-aspartic acid | -1.07 | 0.003594 | M175T74 |
| Stearic acid | -1.06 | 0.015887 | M284T292 |
| Epsilon-caprolactam | -1.15 | 0.020225 | M114T249 |
| Prostaglandin I2 | -1.16 | 0.032641 | M335T368 |
| N-Acetylputrescine | -1.2 | 9.9E-05 | M131T97 |
| Acetylcholine | -1.2 | 0.020286 | M146T51 |
| L-Methionine | -1.17 | 0.000321 | M148T70 |
| Kynurenic acid | -1.27 | 0.000801 | M190T216 |
| 5a-Pregnane-3,20-dione | -1.28 | 0.000384 | M317T587 |
| Arachidonic acid | -1.32 | 0.032581 | M305T556 |
| 3-Epiecdysone | -1.34 | 0.031634 | M464T351 |
| Oxoglutaric acid | -1.35 | 0.032174 | M145T43_1 |
| D-Mannose | -1.39 | 0.015521 | M179T50 |
| Pentadecanoic acid | -1.4 | 0.000272 | M241T620 |
| N-Acetyl-L-phenylalanine | -1.45 | 0.006711 | M208T295 |
| 17-Hydroxyprogesterone | -1.42 | 0.011828 | M331T439 |
| Isotretinoin | -1.48 | 0.003786 | M301T452 |
| Hexadecanedioate | -1.47 | 0.029006 | M285T407 |
| Eugenol | -1.58 | 0.04712 | M165T306 |
| Nonadecanoic acid | -1.59 | 0.017119 | M297T623 |
| Diaminopimelic acid | -1.63 | 0.018259 | M190T241 |
| Aflatoxin B1 | -1.66 | 0.009217 | M312T568 |
| Indole pyruvate | -1.72 | 0.000167 | M204T157 |
| Citric acid | -1.74 | 1.9E-05 | M191T46 |
| N-Formyl-L-methionine | -1.85 | 0.011725 | M176T71 |
| 11alpha,17beta-Dihydroxy-17-methylandrost-4-en-3-one | -1.9 | 0.000247 | M301T420 |
| Pantothenic acid | -1.9 | 0.00253 | M218T71 |
| Imidazol-5-yl-pyruvate | -1.98 | 0.009437 | M155T54 |
| N-Alpha-acetyllysine | -2.03 | 0.007631 | M188T191 |
| beta-D-Fructose 6-phosphate | -2.13 | 0.009772 | M259T44 |
| Indolelactic acid | -2.28 | 0.01683 | M206T304 |
| Antibiotic JI-20B | -2.23 | 0.001067 | M478T490_2 |
| (2R)-2-Hydroxy-3-(phosphonatooxy)propanoate | -2.27 | 7.29E-07 | M167T41 |
| 25-Hydroxycholesterol | -2.44 | 0.020319 | M402T233 |
| UMP | -2.45 | 0.00015 | M323T314 |
| Norethindrone | -2.58 | 0.001595 | M298T328 |
| Daidzein | -2.65 | 0.001067 | M254T338 |
| 5-Amino-2-oxopentanoic acid | -2.71 | 6.93E-05 | M131T72 |
| Trioxilin A3 | -2.87 | 0.002732 | M337T410 |
| Formiminoglutamic acid | -2.91 | 0.003997 | M175T54 |
| N-Acetylserotonin | -3.21 | 0.004549 | M219T245 |
| p-Aminobenzoic acid | -3.64 | 0.027412 | M138T51 |
| Ureidosuccinic acid | -3.76 | 0.000103 | M176T51_2 |
